# Clinical Manifestations of Human Brucellosis: A Systematic Review and Meta-Analysis

**DOI:** 10.1371/journal.pntd.0001929

**Published:** 2012-12-06

**Authors:** Anna S. Dean, Lisa Crump, Helena Greter, Jan Hattendorf, Esther Schelling, Jakob Zinsstag

**Affiliations:** 1 Department of Epidemiology and Public Health, Swiss Tropical and Public Health Institute, Basel, Switzerland; 2 University of Basel, Basel, Switzerland; University of Oklahoma Health Sciences Center, United States of America

## Abstract

**Background:**

The objectives of this systematic review, commissioned by WHO, were to assess the frequency and severity of clinical manifestations of human brucellosis, in view of specifying a disability weight for a DALY calculation.

**Methods/Principal Findings:**

Thirty three databases were searched, with 2,385 articles published between January 1990–June 2010 identified as relating to human brucellosis. Fifty-seven studies were of sufficient quality for data extraction. Pooled proportions of cases with specific clinical manifestations were stratified by age category and sex and analysed using generalized linear mixed models. Data relating to duration of illness and risk factors were also extracted. Severe complications of brucellosis infection were not rare, with 1 case of endocarditis and 4 neurological cases per 100 patients. One in 10 men suffered from epididymo-orchitis. Debilitating conditions such as arthralgia, myalgia and back pain affected around half of the patients (65%, 47% and 45%, respectively). Given that 78% patients had fever, brucellosis poses a diagnostic challenge in malaria-endemic areas. Significant delays in appropriate diagnosis and treatment were the result of health service inadequacies and socioeconomic factors. Based on disability weights from the 2004 Global Burden of Disease Study, a disability weight of 0.150 is proposed as the first informed estimate for chronic, localised brucellosis and 0.190 for acute brucellosis.

**Conclusions:**

This systematic review adds to the understanding of the global burden of brucellosis, one of the most common zoonoses worldwide. The severe, debilitating, and chronic impact of brucellosis is highlighted. Well designed epidemiological studies from regions lacking in data would allow a more complete understanding of the clinical manifestations of disease and exposure risks, and provide further evidence for policy-makers. As this is the first informed estimate of a disability weight for brucellosis, there is a need for further debate amongst brucellosis experts and a consensus to be reached.

## Author Summary

Brucellosis is a bacterial disease transmitted to humans by consumption of infected, unpasteurised animal milk or through direct contact with infected animals, particularly aborted foetuses. The livestock production losses resulting from these abortions have a major economic impact on individuals and communities. Infected people often suffer from a chronic, debilitating illness. This systematic review on the symptoms of human brucellosis is the first ever conducted. Using strict exclusion criteria, 57 scientific articles published between January 1990–June 2010 which included high quality data were identified. Severe complications of brucellosis infection were not rare, with 1 case of endocarditis and 4 neurological cases per 100 patients. One in 10 men suffered from testicular infection, which can case sterility. Debilitating conditions such as joint, muscle, and back pain affected around half of the patients. Given that most patients had fever, brucellosis poses a diagnostic challenge in malaria-endemic areas where fever is often assumed to be malaria. More high quality data is needed for a more complete understanding of the clinical manifestations of disease and exposure risks, and to provide further evidence for policy-makers.

## Introduction

Brucellosis is one of the most common zoonotic infections globally [1]. This bacterial disease causes not only a severely debilitating and disabling illness, but it also has major economic ramifications due to time lost by patients from normal daily activities [2] and losses in animal production [3]. In a review of 76 diseases and syndromes of animals, brucellosis lies within the top ten in terms of impact on impoverished people [4]. A brucellosis disability weighting of 0.2 has been previously proposed for Disability-Adjusted Life Years (DALY) calculation, based on the pain and impaired productivity known to result from infection [3]. However, a more informed estimate is needed for an accurate assessment of disease burden.

In 1992, the World Bank commissioned the original Global Burden of Disease (GBD) study, providing a comprehensive assessment of 107 diseases and injuries and 10 risk factors in eight major regions [5]. This review did not include any neglected tropical zoonoses. Such diseases often do not attract the interest of health researchers or sufficient resources for adequate control, yet they continue to impact significantly on human health and wellbeing, livestock productivity, and local and national economies [6]. There is a need for more accurate data relating to the burden of neglected zoonoses to facilitate more effective implementation of disease control interventions. In 2009, the Foodborne Disease Burden Epidemiology Reference Group (FERG) of the World Health Organization (WHO) commissioned a series of systematic reviews on the burden of neglected zoonotic diseases, with the aim of incorporating the findings into the overall global burden of disease assessments.

This report presents a systematic review of scientific literature published between 1990–June 2010 relating to morbidity from human brucellosis infection. The objectives of this review were to assess the frequency and severity of the clinical manifestations of brucellosis, the duration of disease, the associated disabilities and important risk factors, with a view to estimating an appropriate disability weight for calculation of the brucellosis DALY. A systematic review of scientific literature investigating the incidence and prevalence of brucellosis globally is the subject of a companion paper [7].

## Methods

### Searching

Thirty three databases were searched for relevant articles using the search terms of (brucellosis OR malta fever OR brucella melitensis OR brucella abortus) AND (symptom* OR sequelae* OR morbidity OR mortality OR transmission mode OR foodborne), with a publication limitation of 1990–30 June, 2010. The search term was adapted to the predominate language of the database. If a database did not allow the combining of Boolean operators, (18 of 33 databases), ‘brucellosis’ was used as the sole term.

Reference Manager bibliographic software was used to manage citations. Duplicate entries were identified by considering the author, the year of publication, the title of the article, and the volume, issue and page numbers of the source. In questionable cases, the abstract texts were compared.

### Selection

The articles were sorted by a team of four reviewers with a combined fluency in English, German, French, and Spanish. Articles in other languages were noted for future translation, pending resources.

All reports were classified into one of two categories, based on their abstracts:

Category 1: Relevant – articles related to human brucellosis related to brucellosis infection in populations (i.e. disease frequency) or cases of human brucellosis (i.e. disease morbidity);

Category 2: Irrelevant - articles related to non-human brucellosis; articles addressing topics not related to the current review, such as genetics, laboratory diagnostic tests, experimental laboratory animal studies.

The abstracts of studies belonging to Category 1 and meeting the following criteria for disease morbidity studies were retained: published between 1990 and 30 June 2010, at least 10 study subjects, clinical symptoms/syndromes described, and some information relating to diagnostic tests provided. Articles relating to disease frequency and meeting the following criteria were also retained: published between 1990 and 30 June 2010, at least 100 study subjects drawn from the general population, prevalence or incidence data included, and some information relating to diagnostic tests provided. The assessment and classification of frequency articles will be the subject of a companion paper and will not be considered further here.

Articles for which the necessary data for classification could not be obtained were identified for possible future assessment, according to availability of resources. In general, non peer-reviewed or review articles, conference proceedings and book chapters were excluded.

### Validity Assessment

After applying the aforementioned screening steps, the full text of each selected article was retrieved for detailed analysis. Each article was reviewed by two or three reviewers, and classification discrepancies were resolved by discussion.

Using a pre-designed Access database, articles were coded according to the following parameters:

1) Study type

Studies were classified as a prospective case series, a retrospective case series, a case-control study, or of another type.

2) Study population

The populations studied were grouped according to age category – children only (<15 years), adults only (≥15 years), or including both children and adults. Additionally, they were coded according to whether the study population represented the general population of brucellosis cases in the age category, or only a specific sub-group.

3) Diagnostic methods

Studies were classified according to their use of microbial culture to diagnose brucellosis patients. In order for studies to be included in the review, they had to not only mention culture in their methods but to also present laboratory results.

4) Overall study quality

Studies were given an overall quality grade of 1, 2, or 3. Quality 1 studies provided data drawn from general brucellosis cases, of which 75% or more were diagnosed by culture, and had well described study design and methods. Quality 2 studies also presented data from general brucellosis cases, utilised culture as a method and presented relevant laboratory results. However, unlike for Quality 1 studies, the majority of cases did not have to be diagnosed by positive culture in order to be included as Quality 2. Quality 3 studies were either drawn from only a specific sub-group of brucellosis cases such that general conclusions could not be drawn, did not use culture as a diagnostic method or failed to present culture results, or had poorly described study design and methods such that the quality of the data could not be assured.

### Data Extraction

Based on brucellosis literature [8] a comprehensive list of clinical manifestations associated with brucellosis cases was developed:

General: documented fever, sweats, chills, fatigue, headache, malaise, weight loss, nausea/vomiting
Abdominal: abdominal pain, splenomegaly, hepatomegaly, hepatitis
Musculoskeletal: arthralgia, arthritis, myalgia, back pain, spondylitis, sacroiliitis
Specific organ involvement: epididymo-orchitis, abortion, endocarditis, respiratory and neurological signs, cutaneous changes

Numbers of subjects with each symptom/syndrome were recorded for each study, as well as the number of male and female patients. For the sex-related outcomes of epididymo-orchitis and abortion, the study population was considered to be only the male and pregnant female sub-groups of the study population respectively. Information relating to duration of disease prior to treatment and exposure to potential risk factors were also recorded wherever provided.

### Data Analysis

To calculate the proportion of patients by sex, numbers of male and female patients were aggregated across all studies as well as within each age category. 95% confidence intervals were calculated using the normal approximation to the binomial.

Where appropriate data were available from two or more studies, pooled proportions of patients with each clinical manifestation were estimated using generalized linear mixed models. Pooled estimates with 95% confidence intervals were calculated both within age categories and overall across all studies, using a Freeman-Tukey double arscine transformation. Homogeneity across studies was assessed using a Cochrane's Q test and total variability due to between-study variation was reflected in the I^2^ index. The meta-analysis was performed with R statistical software [9] using the meta package [10]. Additionally, in order to assess the impact of study design, the same analysis was conducted according to study type category.

The pooled estimates for proportions of patients with each clinical manifestation were compared with the disability weights used in the GBD 2004 study [11]. A disability weight for brucellosis was then proposed.

Median proportions of patients with exposure to particular risk factors were calculated. Data relating to duration of illness and diagnostic delay were recorded. In order to assess the duration of untreated illness, an additional, non-systematic search for data prior to the availability of appropriate antibiotics was undertaken by manually searching library records.

## Results

### Searching

Table 1 lists the databases searched and the number of hits obtained for each. A total of 28,824 studies were identified, of which 59% were duplicates, leaving 11,000 original reports.

**Table 1:** Databases searched and number of hits.

Database	Website	Hits
***Global databases***		
Medline	http://www.ncbi.nlm.nih.gov/sites/pubmed	6176
ISI Web of Science	http://isiwebofknowledge.com	3458
EMBASE	http://www.embase.com	4980
Popline	http://www.popline.org	55
CAB	http://www.cabdirect.org	3424
ProMed	http://www.promedmail.org	666
The Cochrane Library	http://www.thecochranelibrary.com	100
BIOLINE	http://www.bioline.org.br	37
WHOLIS	http://www.bireme.br	76
***Regional WHO databases***		
African Index Medicus	http://indexmedicus.afro.who.int	14
Index Medicus for the Eastern Mediterranean Region	http://www.emro.who.int/whalecom0/Library/Databases/wxis.exe/Library/Databases/iah/	526
Western Pacific Region Index Medicus	http://www.wprim.org/	96
Index Medicus for the South-East Asia Region	http://imsear.hellis.org/	247
Afro Library	http://afrolib.afro.who.int/	2
***Other regional databases***		
Health Information Locator	http://www.bireme.br	7
Institute of Tropical Medicine, Antwerp, Belgium	http://lib.itg.be:8000/webspirs/start.ws	122
King's Fund Information & Library Service	http://www.kingsfund.org.uk/library/	0
African Journals Online	http://ajol.info/	71
LILACS	http://www.bireme.br	538
MedCarib	http://www.bireme.br	9
REPIDISCA	http://www.bireme.br	29
PAHO	http://www.bireme.br	157
IBECS	http://www.bireme.br	148
CUIDEN	http://www.index-f.com/	17
Indian Medlars Center IndMed	http://indmed.nic.in/	84
KoreaMed	http://www.koreamed.org/SearchBasic.php	89
Japan Science and Technology Information Aggregator	http://www.jstage.jst.go.jp/search/?typej=on&typep=on&typer=on&search=1	137
Health Research and Development Information Network	http://www.herdin.ph/	0
Panteleimon	http://www.panteleimon.org/maine.php3	6
l'Ecole Nationale de la Santé Publique	http://test.bdsp.ehesp.fr/Base/	191
La Bibliotàgue de Santé Tropicale	http://www.santetropicale.com/resume/catalogue.asp	0
System for Information on Grey Literature in Europe	http://opensigle.inist.fr	474
Swiss Tropical and Public Health Institute, Human and Animal Health Unit, electronic departmental reference library		6906

### Flow of Included Studies

**Figure 1:**
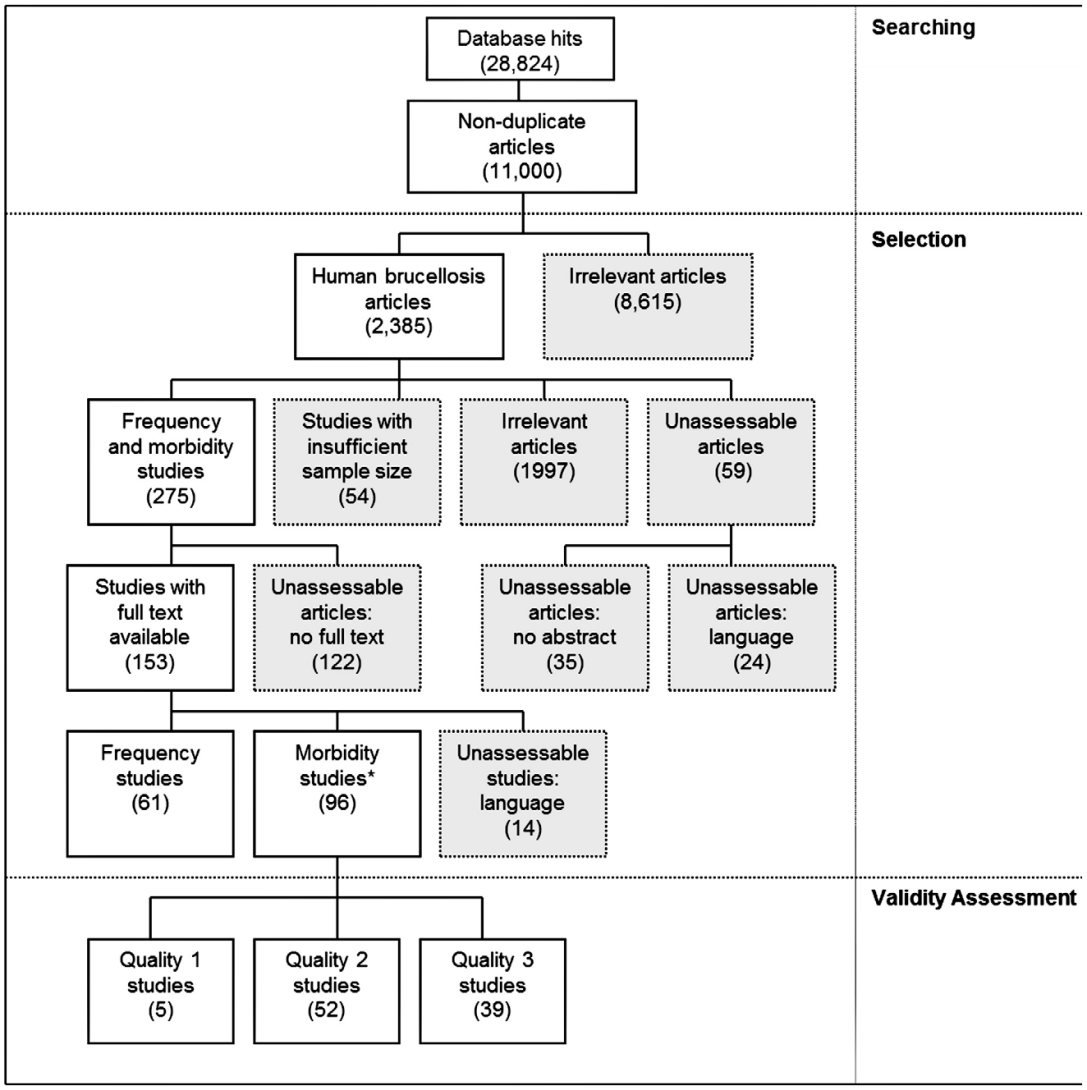
Flow of selected studies. *Some morbidity studies were also classified as frequency studies.

Figure 1 shows a flow diagram of the process for the selection of articles included in the review. In total, 289 frequency and morbidity studies were selected, for which full text was available for 153. However, 14 of these were in languages in which the team was not competent (Croatian (6), Turkish (4), Korean (2), Persian (1), Mandarin (1)), leaving 96 morbidity studies for quality assessment. Some articles contained both frequency and morbidity data and were thus counted in both categories.

Of the 96 morbidity studies for quality assessment, five were classified as Quality 1 and 52 as Quality 2. Thirty-nine were excluded from further analysis as Quality 3, one of which was due to duplication of data from another larger study. Two pairs of Quality 2 studies were based on the same data [12]–[15]. These studies were included because each provided some unique information; however, the duplicated data were only included once in the meta-analysis. Except for two articles in Spanish and one in French, all Quality 1 and 2 studies were in English.

### Study Characteristics

**Figure 2:**
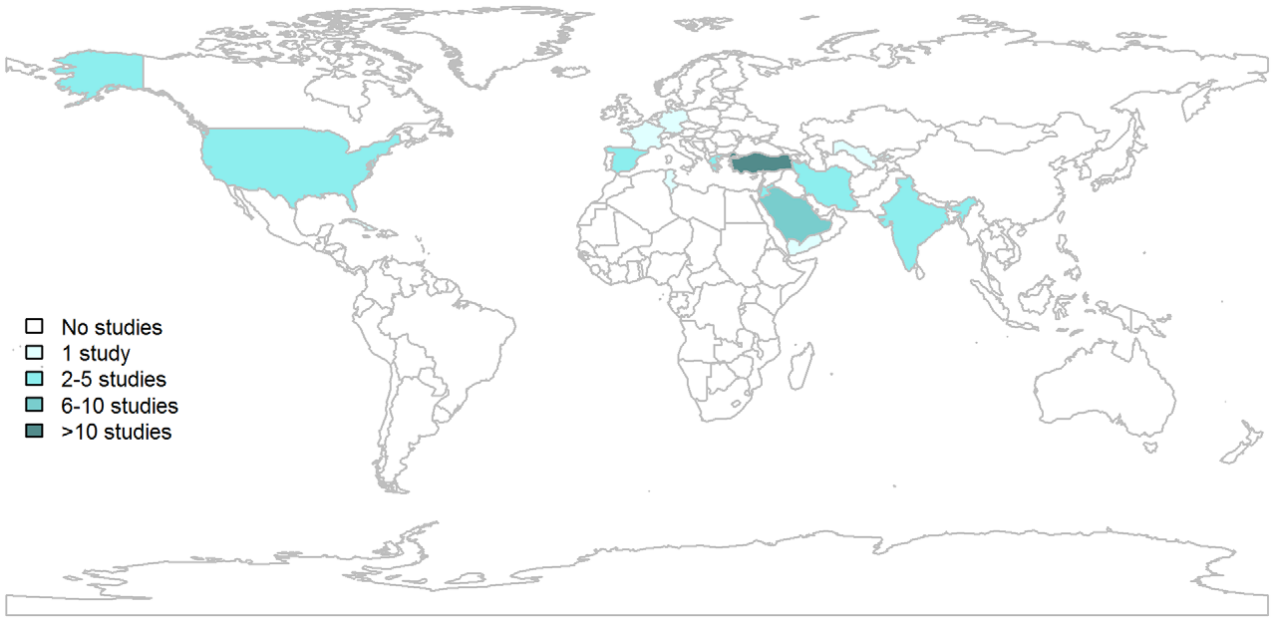
Geographical distribution of selected studies.

The median number of study subjects was 143 (IQR: 85-283), ranging from 20-1028. Studies from high income countries such as Germany, France, and USA were generally situated at the lower end of the range (less than 60 subjects), although larger studies were reported from Spain, including one study of over 900 subjects. Of the 57 studies selected, 24 were from Turkey. The next most represented country was Saudi Arabia, with 8 studies, followed by Spain with 4 and Greece with 4. One or two studies each came from Cuba, France, Germany, Israel, India, Iran, Jordan, Kuwait, Tunisia, USA, Uzbekistan and Yemen. The geographic distribution of the selected studies is shown in Figure 2.

In terms of study type, 37 were classified as retrospective case series with data retrieved from medical records, and 19 as prospective case series. One study was a case-control. Seventeen studies provided detailed information about cases with specific syndromes, e.g. neurological brucellosis [16]–[19], epididymoorchitis [20]–[23], osteoarticular complications [13], [14], [24], [25], spondylitis [26], [27], pulmonary brucellosis [28], pancytopaenia [29], and pregnant women [30]. As these studies also provided some information about proportions of general brucellosis cases with specific symptoms/syndromes, they were included in the review.

Twenty-three studies included both children and adult participants [12]–[15], [18], [20], [24], [30]–[44]. Twelve studies investigated only children [29], [45]–[55], with an upper age limit ranging from 13 years to 18 years. Of the 19 studies with an adult population of 15 years or older [16], [17], [21]–[23], [25]–[27], [56]–[67], five consisted of only male participants [21]–[23], [64], [65]. Three studies did not clearly state the age category [19], [28], [68] and were analysed as if containing data for both adults and children.

### Data Analysis

In studies consisting of only children, 64% patients (95% CI: 60–68%) were male. The proportion of male patients in adult studies was significantly lower, at 56% (95% CI: 55–58%). In studies including both children and adult patients, 48% were male (95% CI: 46–51%). Overall, 55% patients (95% CI: 54–56%) across all studies were male.

Table 2 shows the pooled proportions of patients estimated by the random-effects model, according to clinical manifestations by age category. Forest plots are provided as Supplementary Information. An analysis by study type did not show any significant changes or trends.

**Table 2:** Meta-analysis of clinical manifestations of brucellosis by age category.

Manifestation	Age Category						All studies	
	Children		Adults		All Ages			
*General*	*n*	*% (95% CI)*	*n*	*% (95% CI)*	*n*	*% (95% CI)*	*n*	*% (95% CI)*
Fever	7	82 (69; 91)	10	73 (59; 85)	9	79 (49; 97)	26	78 (66; 87)
Sweats	8	23 (11; 37)	14	55 (35; 74)	12	73 (60; 85)	34	54 (42; 66)
Chills	4	18 (9; 29)	5	47 (34; 60)	7	60 (34; 83)	16	45 (30; 61)
Fatigue	2	19 (13; 23)	2	33 (13; 100)	5	51 (27; 75)	9	39 (16; 65)
Headache	6	9 (5; 15)	11	34 (19; 50)	11	52 (32; 72)	28	35 (24; 46)
Malaise	2	24 (16; 34)	6	81 (71; 89)	8	74 (48; 93)	16	71 (57; 83)
Nausea/vomiting	0	-	5	16 (5; 31)	6	26 (15; 38)	11	26 (15; 38)
Weight loss	3	13(8;18)	4	31 (15; 50)	7	29 (15; 47)	14	26 (17; 36)
*Abdominal*								
Abdominal pain	3	14 (1; 38)	4	9 (1; 22)	9	26 (13; 41)	16	19 (11; 29)
Splenomegaly	9	31 (19; 43)	13	24 (18; 31)	14	25 (17; 34)	36	26 (21; 31)
Hepatomegaly	10	27 (15; 41)	13	22 (16; 26)	14	22 (15; 29)	37	23 (19; 27)
Hepatitis	1	1 (0; 5)*	2	8 (1; 38)	4	3 (1; 6)	7	4 (1; 9)
*Musculoskeletal*								
Arthralgia	9	71 (56; 84)	12	65 (49; 79)	16	62 (52; 70)	37	65 (58; 72)
Arthritis	7	41 (18; 65)	5	13 (3; 28)	14	25 (17; 34)	26	26 (19; 34)
Myalgia	2	18 (11; 26)	5	56 (38; 75)	8	49 (36; 63)	15	47 (38; 57)
Back pain	1	10 (3; 21)*	11	49 (31; 67)	11	45 (31; 60)	23	45 (34; 56)
Sacroiliitis	4	6 (3; 10)	3	32 (20; 46)	9	14 (7; 22)	16	15 (9; 22)
Spondylitis	1	18 (1; 28)*	6	12 (7, 19)	9	11 (6; 18)	16	12 (8; 17)
*Specific organs*								
Epididymo-orchitis	1	10 (1; 32)*	10	10 (7; 15)	10	9 (6; 13)	21	10 (7; 13)
Endocarditis	2	3 (1; 6)	6	2 (1; 3)	7	1 (1; 2)	15	2 (1; 2)
Neurological	5	2 (1; 4)	11	5 (3; 7)	10	4 (2; 6)	26	4 (3; 5)
Respiratory	3	5 (1; 14)	5	2 (1; 5)	11	9 (4; 14)	19	6 (3; 9)
Cutaneous	6	5 (2; 10)	4	4 (1; 11)	7	8 (4; 14)	17	6 (4; 9)

* **One study only, with a binomial 95% confidence interval.**

Pooled proportions of patients with each manifestation are presented as percentages with 95% confidence intervals. The numbers of studies (*n*) contributing to each estimate are given.

Documented fever was common, with an estimated 78% of patients affected across the three age categories. Estimates of the proportions of patients with self-reported symptoms of sweats, chills, fatigue, headache, and malaise, were significantly lower in children, ranging from 9–24% depending on symptom, compared to 33–81% for adults. Weight loss in children, at 13%, was also lower than the 31% reported in adults.

Abdominal-related manifestations of pain, splenomegaly and hepatomegaly were fairly uniformly distributed across age categories, with overall estimated proportions of 19%, 26% and 23%, respectively. The number of studies reporting the presence of hepatitis was small, totalling only seven, with an estimated 4% patients affected overall.

Arthralgia was common, affecting 65% patients overall, whereas arthritis affected only 26% patients. In adult patients, 56% and 49% suffered from myalgia and back pain, respectively. Only two studies reported myalgia and back pain in children. Overall, spondylitis and sacroiliitis were detected in 12–36% adults.

In relation to reproductive problems, only one study reported abortion rates as a proportion of pregnant female participants, which was 46% [30]. Overall, 10% male patients had epididymo-orchitis.

For more severe outcomes, endocarditis was reported in an overall 1% patients, and neurological manifestations in 4%. Neurological outcomes reported included motor deficits, cranial nerve deficits, sciatica, confusion and/or psychological disturbances, meningitis and seizures. 6% of patients suffered from respiratory manifestations, including cough, bronchopneumonia, pleural adhesion and pleural adhesion. Cutaneous changes were reported in 6% patients.

As most studies were case series without a control group, an evaluation of the importance of risk factors was not possible. However, median proportions were calculated from 27 studies which provided some exposure history. Median proportions of brucellosis cases with exposure to a potential risk factor were 64% (IQR: 34–78%) for consumption of unpasteurised dairy products, 42% (IQR: 23–59%) for contact with livestock, and 6% (IQR: 3–19%) for occupational exposure, including veterinarians, butchers, and abattoir workers. From fifteen studies, the median proportion of cases with a history of brucellosis in a family member was 20% (IQR: 17–46%).

Only six studies included in the systematic review provided data regarding duration of illness prior to diagnosis and treatment [32], [41], [52], [55], [57], [62]. The age of the patient and the nature of the illness were influential factors. One study reported a longer duration of illness in adults compared to children under 15 years, averaging 8 weeks versus 4 weeks, respectively [41]. In another study, the average duration of illness prior to diagnosis and treatment was 40 days, but cases with osteoarticular disease generally experienced longer periods of illness, extending to 6 months [62].

The GBD 2004 study estimated the disability weights for low back pain due to chronic intervertebral disc disease and osteoarthritis of the knee to be 0.121 (range 0.103–0.125) and 0.129 (range 0.118–0.147), respectively [11]. Given the high proportion of patients in our systematic review with joint, back, or muscular pain, a disability weight of at least 0.150 is proposed as a minimum estimate for localised, chronic brucellosis. Generalised, non-specific clinical manifestations were also common. Acute, non-localised brucellosis could be approximated by an episode of malaria, estimated to be 0.191 (range 0.172–0.211) by the GBD 2004 study [11].

## Discussion

The clinical picture of brucellosis presented in this systematic review is consistent with other literature [69]. Although a large amount of data are available regarding clinical manifestations of brucellosis, its geographical distribution is limited. No high quality studies were identified from Sub-Saharan Africa, Central and South America or South-East Asia. This could potentially reflect either a lower disease burden or a poorer brucellosis surveillance system.

The proportion of male patients was greater than female patients amongst both children and adults. Although this difference was only small in adults, it was more pronounced in children. Possible explanations could be a greater risk of exposure amongst boys, with household responsibilities such as shepherding of livestock being preferentially delegated to boys, or gender-related differences in accessing to health care.

Given the high proportion of brucellosis cases with fever, brucellosis should be considered as a differential diagnosis for fevers of unknown origin. In malaria-endemic countries, fever patients are often diagnosed and treated for malaria based solely on clinical findings [70]. Improved diagnostic capacity would reduce the diagnostic delay and facilitate prompt and appropriate treatment. These health service inadequacies are compounded by socioeconomic factors, with brucellosis affecting poor, marginalised communities who often do not have the means to seek treatment. Although studies included in this systematic review did not investigate health-seeking behaviour, a study from rural Tanzania revealed that 1 in 5 patients did not present to a health centre for assessment until more than one year after the onset of illness. Once at the health centre, nearly half (45%) were not diagnosed with brucellosis at their first visit [71]. In children, particularly, under-diagnosis of brucellosis is likely. The lower proportions of reported general symptoms such as sweats, chills, fatigue, and headache in study populations consisting only of children in this systematic review could reflect difficulty in obtaining accurate case histories from this group.

One in 10 men experienced epididymo-orchitis, the most common genitourinary complication of brucellosis infection. This can have serious repercussions such as abscessation and infertility. Although other severe outcomes were less common, 4 neurological cases and 1 endocarditis case per 100 brucellosis patients were reported, which is substantial.

Arthralgia, myalgia, and back pain were common manifestations. The relative lower proportions of patients with sacroiliitis and spondylitis compared to those reporting back pain might reflect limitations in diagnostic capacity. Chronic pain has been shown to severely affect the quality of sufferers' social and working lives [72]. As the majority of the brucellosis disease burden is in less developed countries, where livelihoods are often reliant on physical activities, the impact of musculoskeletal pain and impaired function in these settings may be even more serious.

One study reported that patients with osteoarticular disease experienced a greater diagnostic delay than other cases [62], reflecting the chronic debilitation that can result from brucellosis infection. Indeed, in an endemic area of Russia prior to the availability of effective antibiotic therapies approximately 40% of 1,000 brucellosis cases followed over a 20 year period continued to suffer from clinical manifestations two years after disease onset. In this study, cited by Wund in 1966, approximately 90% of cases had self-cured after 6 years. [73].

Given the complexity of the clinical manifestations of brucellosis, summarising its impact into a single disability weight risks being too reductionist. However, a disability weight is required for an assessment of the global burden of disease which is, in turn, essential for engagement of policy-makers and funding bodies. Using the disability classes formerly used by the GBD 2004 study [74], a disability weight of 0.2 has been previously proposed based on Mongolian patient data [3]. This estimate fell between Class 1 (0.096), which referred to a limited ability to perform at least one activity in the one of the following areas: recreation, education, procreation or occupation; and Class 2 (0.22), referring to a limited ability to perform most activities in one of the aforementioned areas.

Based on this systematic review and meta-analysis, better informed estimates of disability weights are proposed: at least 0.150 for chronic, localised brucellosis and 0.190 for acute brucellosis. However, as this is the first informed estimate of a brucellosis disability weight, there is a need for further debate amongst brucellosis experts and a consensus to be reached.

### Research Agenda

Morbidity could vary geographically according to epidemiological setting. Well designed epidemiological studies from regions under-represented in this review would greatly contribute to an overall assessment of the global disease burden. A surveillance system amongst fever patients in malaria-endemic countries could be particularly informative. Additionally, risk factors for disease should be investigated through case-control studies. This would provide invaluable information to guide disease control interventions and policy.

### Limitations

Studies for which a title or abstract was not published in a language using the Latin alphabet, such as those published only in Chinese characters or Arabic script, may not have been identified during the original database search. Of the foreign language studies that were identified, those published in languages in which the team was not competent were excluded from the analysis. It is possible that some of these studies contained data that could have contributed to this global assessment of brucellosis morbidity. Additionally, although studies in English were independently reviewed by three team members, this was not always possible for studies reviewed in other languages (German, French, Spanish).

There were likely some differences between the case definitions and diagnostic capacity of different studies. For neurological and respiratory syndromes, many studies provided only an overall aggregated estimate without details of the different disease forms. A respiratory case could potentially vary from a patient with only a cough to severe bronchopneumonia, or a neurological case from altered behaviour and confusion to nerve deficits, meningitis or seizures. All patients were positive by culture in only 3 studies. Given the complexity of brucellosis serology interpretation, it is possible that some patients in other studies were misdiagnosed as cases of active brucellosis.

The studies provide data from brucellosis patients presenting to health centres. It is possible that cases that do not present to health centres are less severe. The results of this review may, therefore, be biased towards more severe cases. As with the estimation of other disability weights, the proposed brucellosis disability weight estimate assumes that a given clinical manifestation will result in the same disability in all settings, which is unlikely [75].

### Conclusion

This systematic review adds to the understanding of the global burden of brucellosis, one of the most common and important zoonotic diseases worldwide. Brucellosis is shown to have a severe, debilitating, and often chronic impact on its sufferers. Significant delays in appropriate diagnosis and treatment are the result of both health system inadequacies and socioeconomic factors. Well designed epidemiological studies from those regions identified to be lacking in data would allow a better understanding of the clinical manifestations of disease and exposure risks and provide further evidence for policy-makers. Based on the findings of this systematic review and the disability weights from the 2004 Global Burden of Disease Study, a disability weight of 0.150 is proposed as the first informed estimate for chronic, localised brucellosis and 0.190 for acute brucellosis. As this is the first informed estimate of a disability weight for brucellosis, there is a need for further debate amongst brucellosis experts and a consensus to be reached.

## Supporting Information

Checklist S1 **PRISMA checklist.**
(DOC)

Figure S1 **Forest plot for fever.**
(TIFF)

Figure S2 **Forest plot for sweats.**
(TIFF)

Figure S3 **Forest plot for chills.**
(TIFF)

Figure S4 **Forest plot for fatigue.**
(TIFF)

Figure S5 **Forest plot for headache.**
(TIFF)

Figure S6 **Forest plot for malaise.**
(TIFF)

Figure S7 **Forest plot for nausea/vomiting.**
(TIFF)

Figure S8 **Forest plot for weight loss.**
(TIFF)

Figure S9 **Forest plot for abdominal pain.**
(TIFF)

Figure S10 **Forest plot for splenomegaly.**
(TIFF)

Figure S11 **Forest plot for hepatomegaly.**
(TIFF)

Figure S12 **Forest plot for hepatitis.**
(TIFF)

Figure S13 **Forest plot for arthralgia.**
(TIFF)

Figure S14 **Forest plot for arthritis.**
(TIFF)

Figure S15 **Forest plot for myalgia.**
(TIFF)

Figure S16 **Forest plot for back pain.**
(TIFF)

Figure S17 **Forest plot for sacroiliitis.**
(TIFF)

Figure S18 **Forest plot for spondylitis.**
(TIFF)

Figure S19 **Forest plot for epididymo-orchitis.**
(TIFF)

Figure S20 **Forest plot for endocarditis.**
(TIFF)

Figure S21 **Forest plot for neurological sequelae.**
(TIFF)

Figure S22 **Forest plot for respiratory sequelae.**
(TIFF)

Figure S23 **Forest plot for cutaneous sequelae.**
(TIFF)
