# Supplementary figures and images for: Clinical Manifestations of Human Brucellosis: A Systematic Review and Meta-Analysis
Source: PLoS Negl Trop Dis. 2012 Dec 6;6(12):e1929. doi: 10.1371/journal.pntd.0001929 (PMC3516581; doi:10.1371/journal.pntd.0001929)

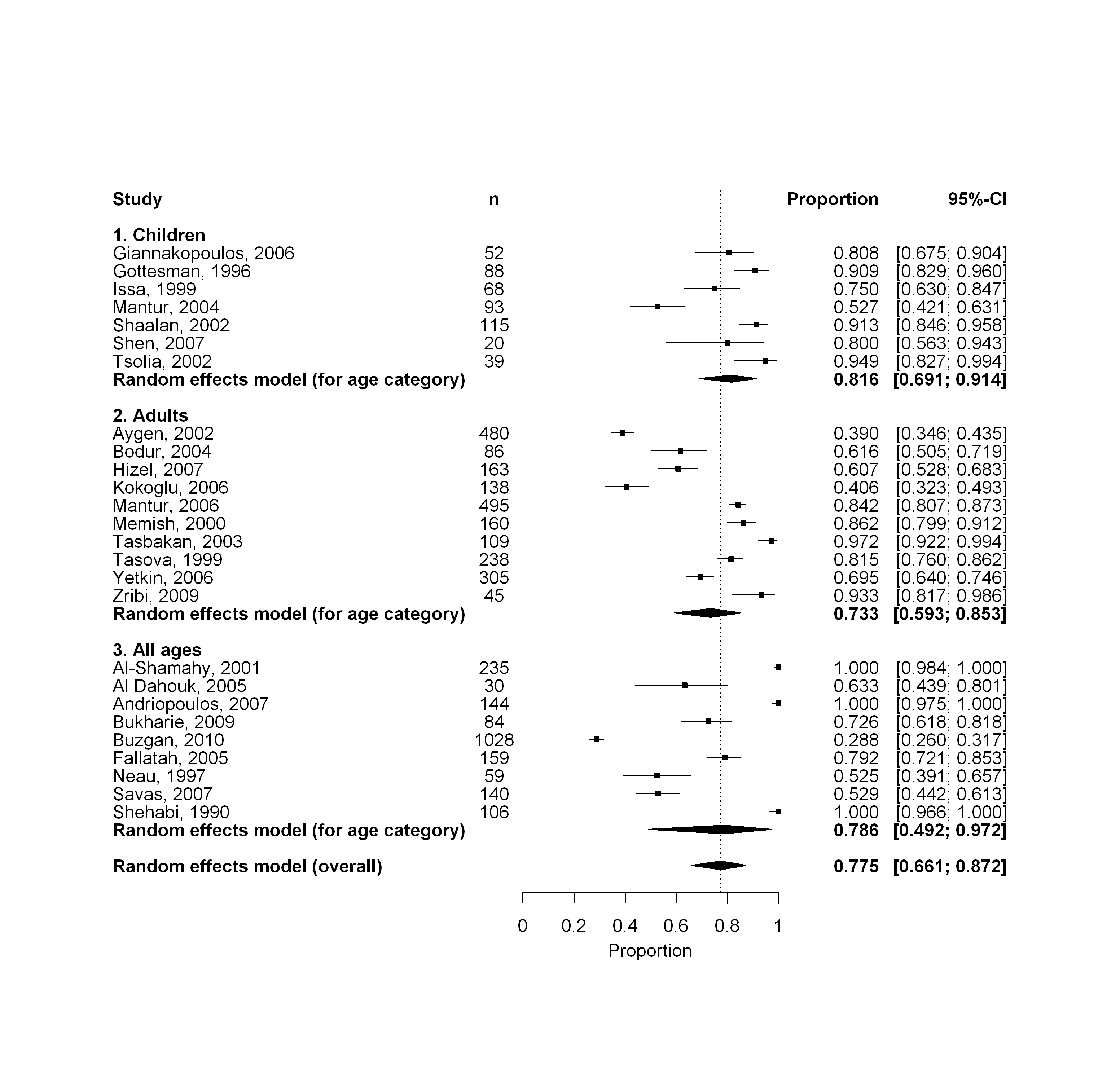

Supplement: Figure S1 — Forest plot for fever. (TIFF) [file pntd.0001929.s002.tiff]

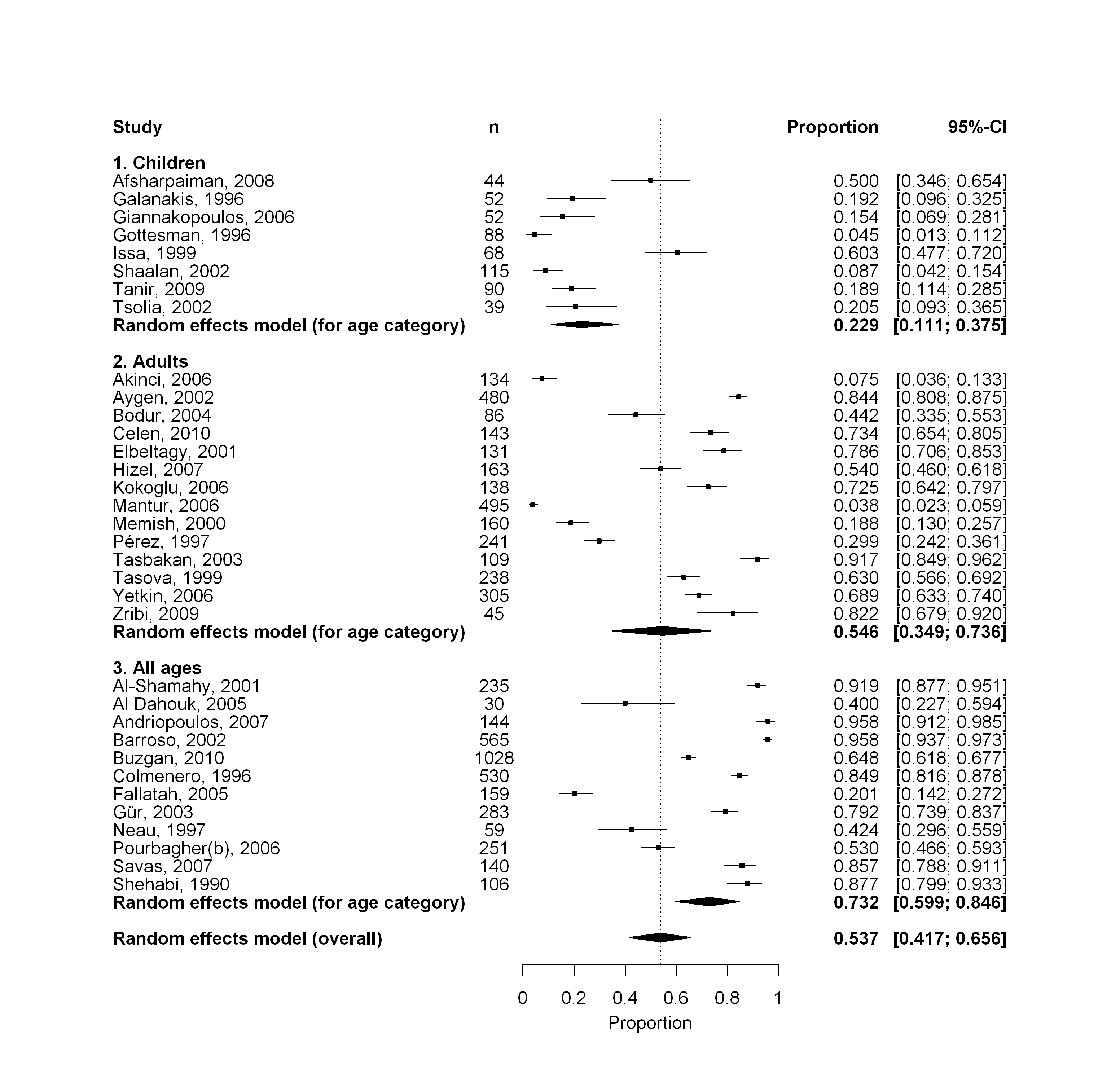

Supplement: Figure S2 — Forest plot for sweats. (TIFF) [file pntd.0001929.s003.tiff]

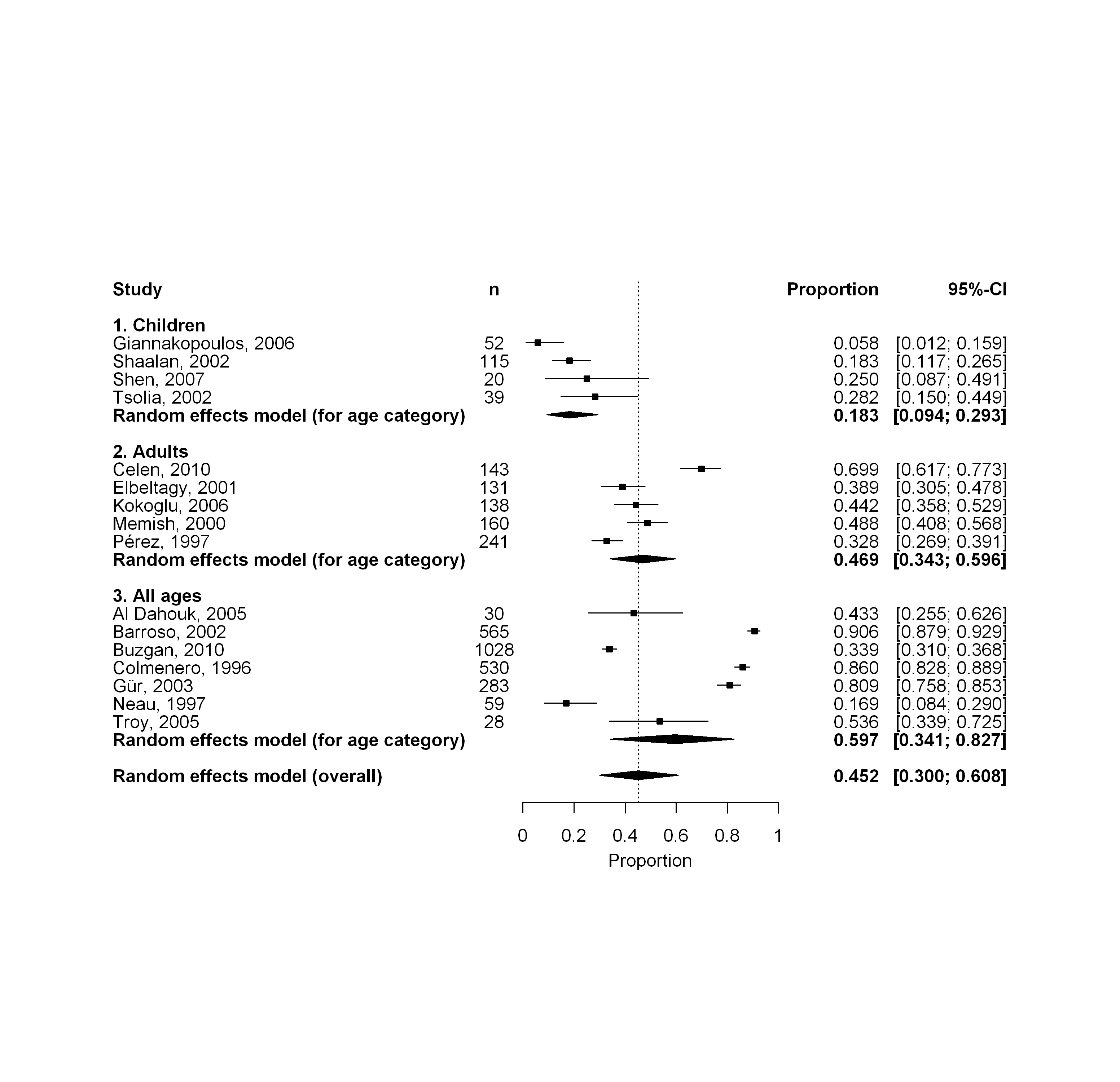

Supplement: Figure S3 — Forest plot for chills. (TIFF) [file pntd.0001929.s004.tiff]

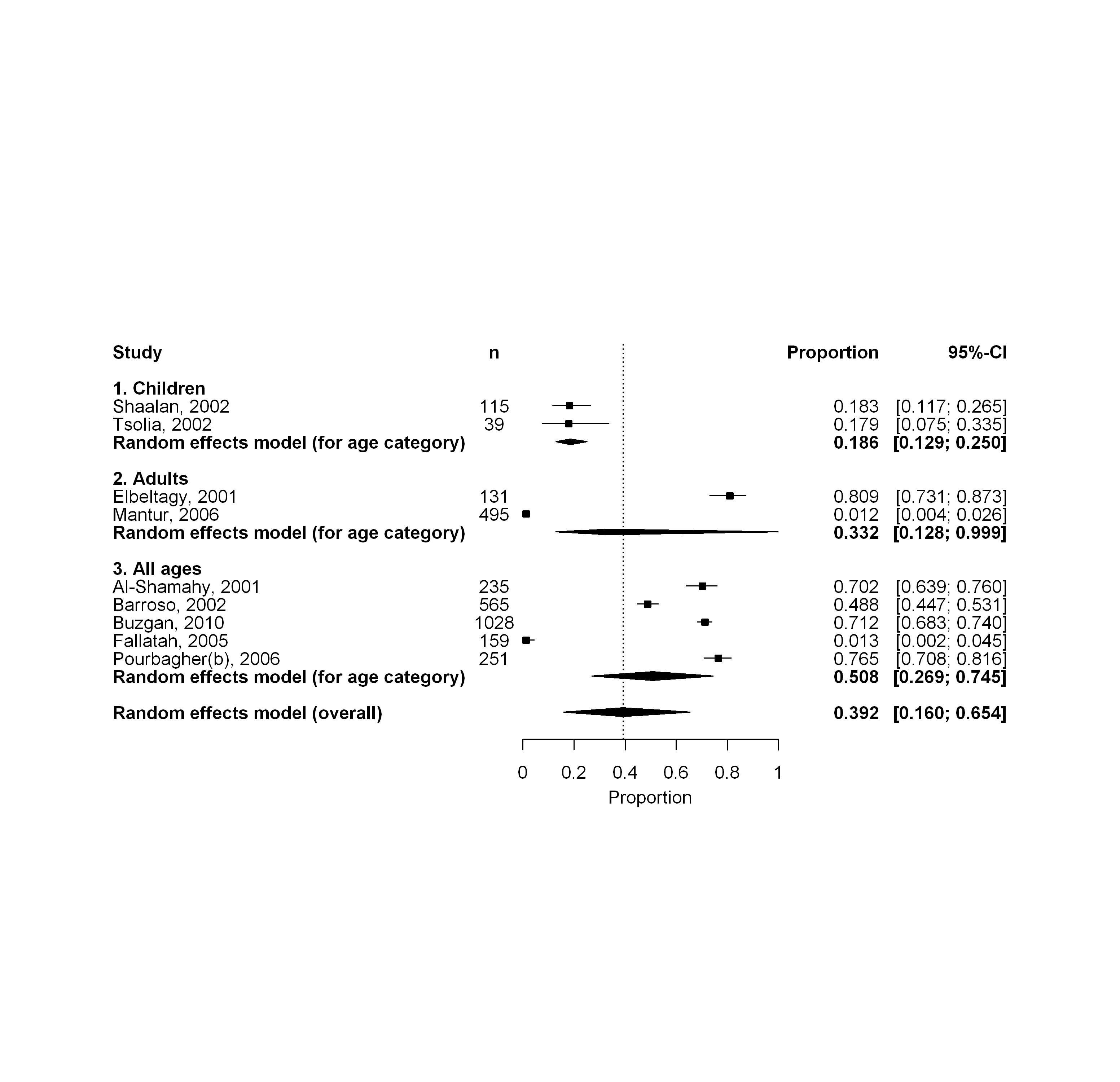

Supplement: Figure S4 — Forest plot for fatigue. (TIFF) [file pntd.0001929.s005.tiff]

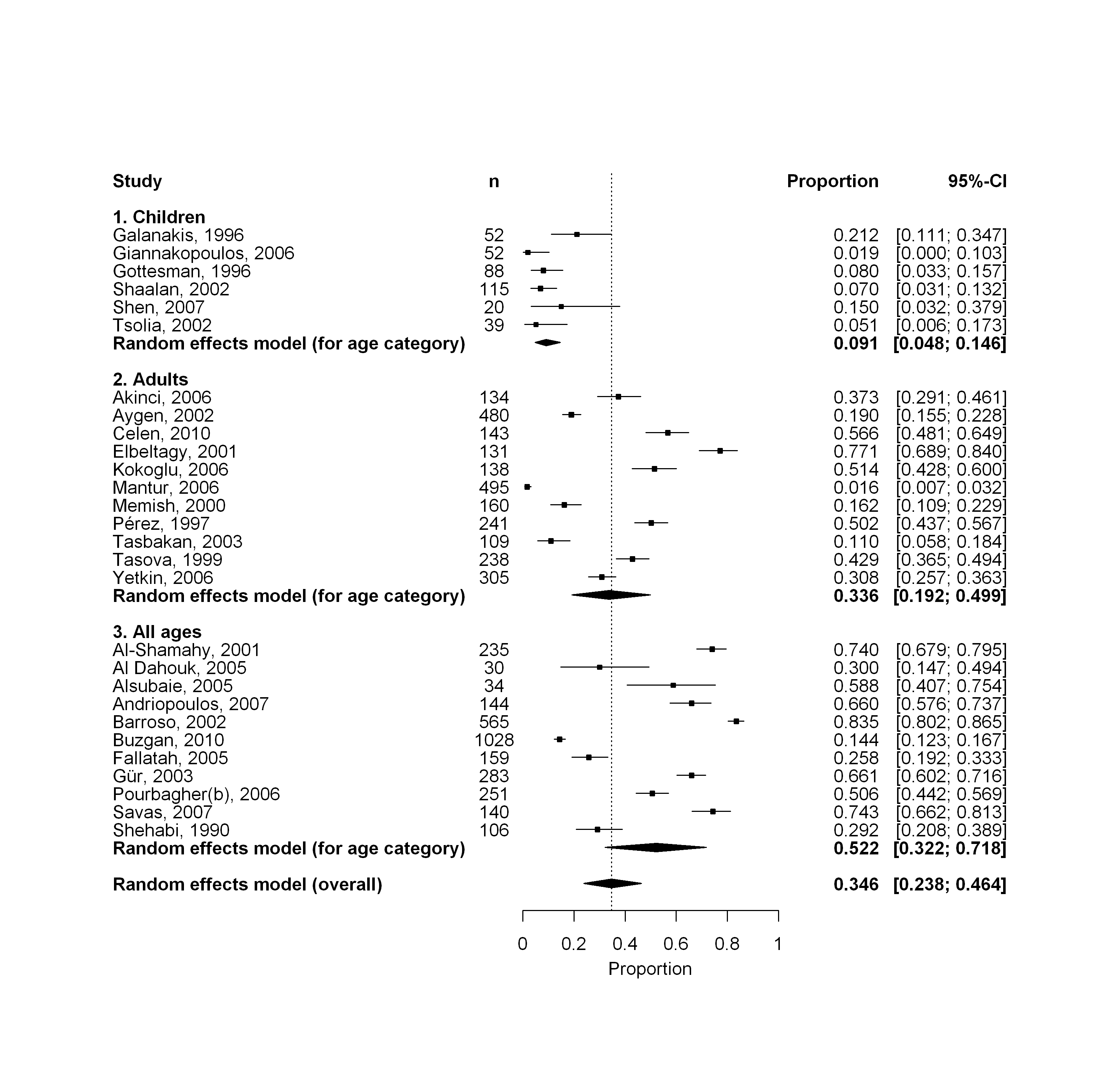

Supplement: Figure S5 — Forest plot for headache. (TIFF) [file pntd.0001929.s006.tiff]

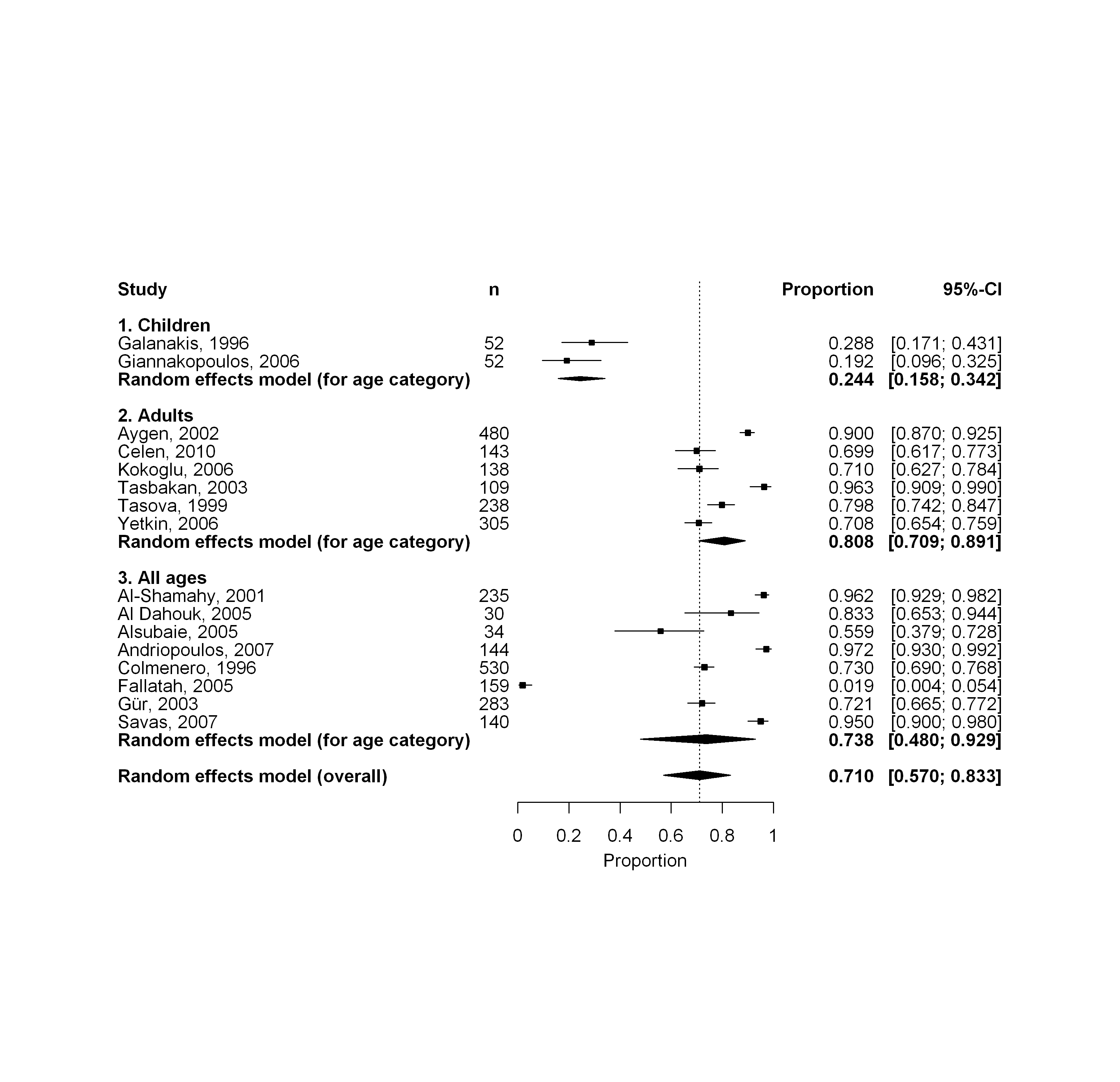

Supplement: Figure S6 — Forest plot for malaise. (TIFF) [file pntd.0001929.s007.tiff]

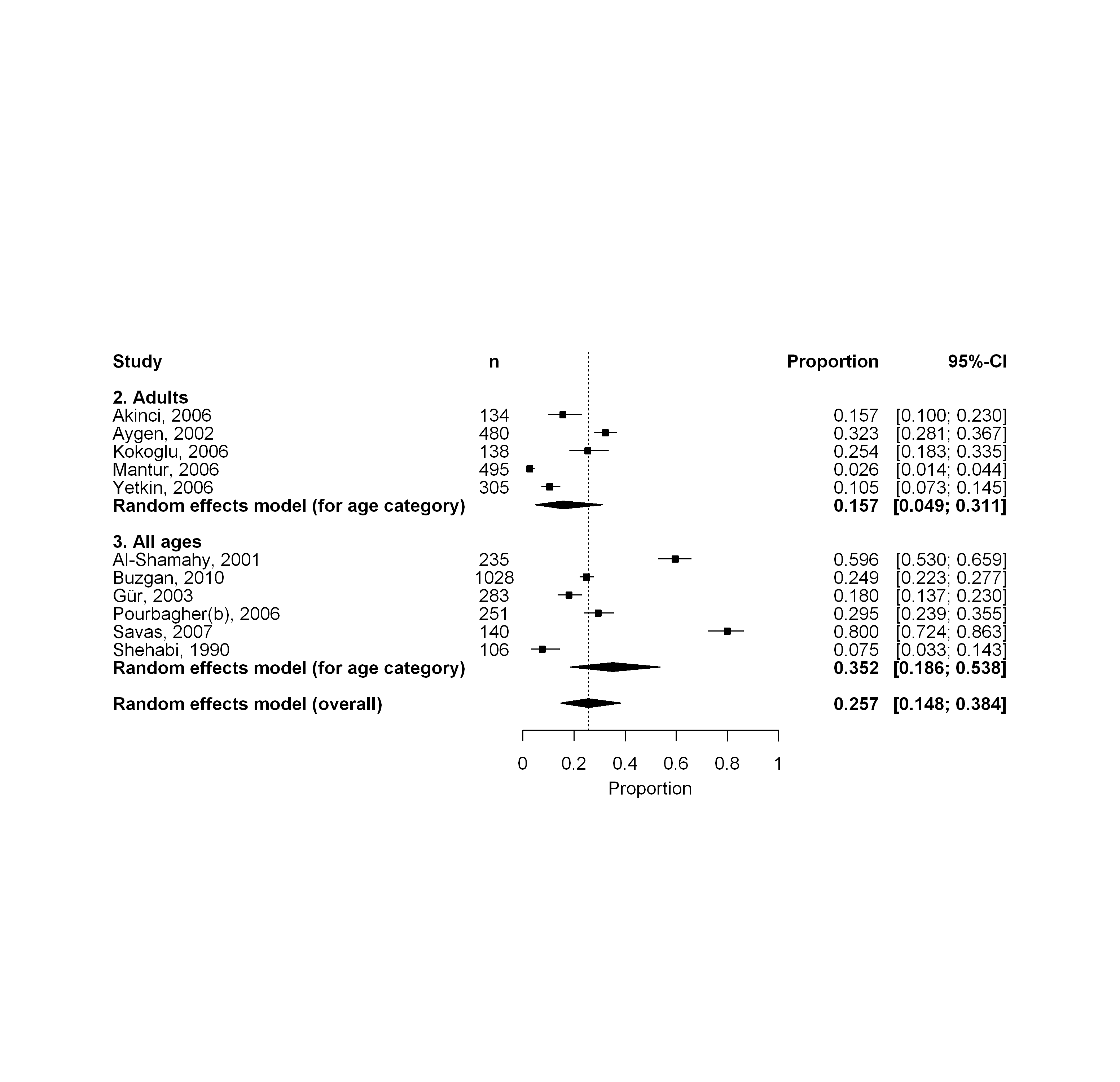

Supplement: Figure S7 — Forest plot for nausea/vomiting. (TIFF) [file pntd.0001929.s008.tiff]

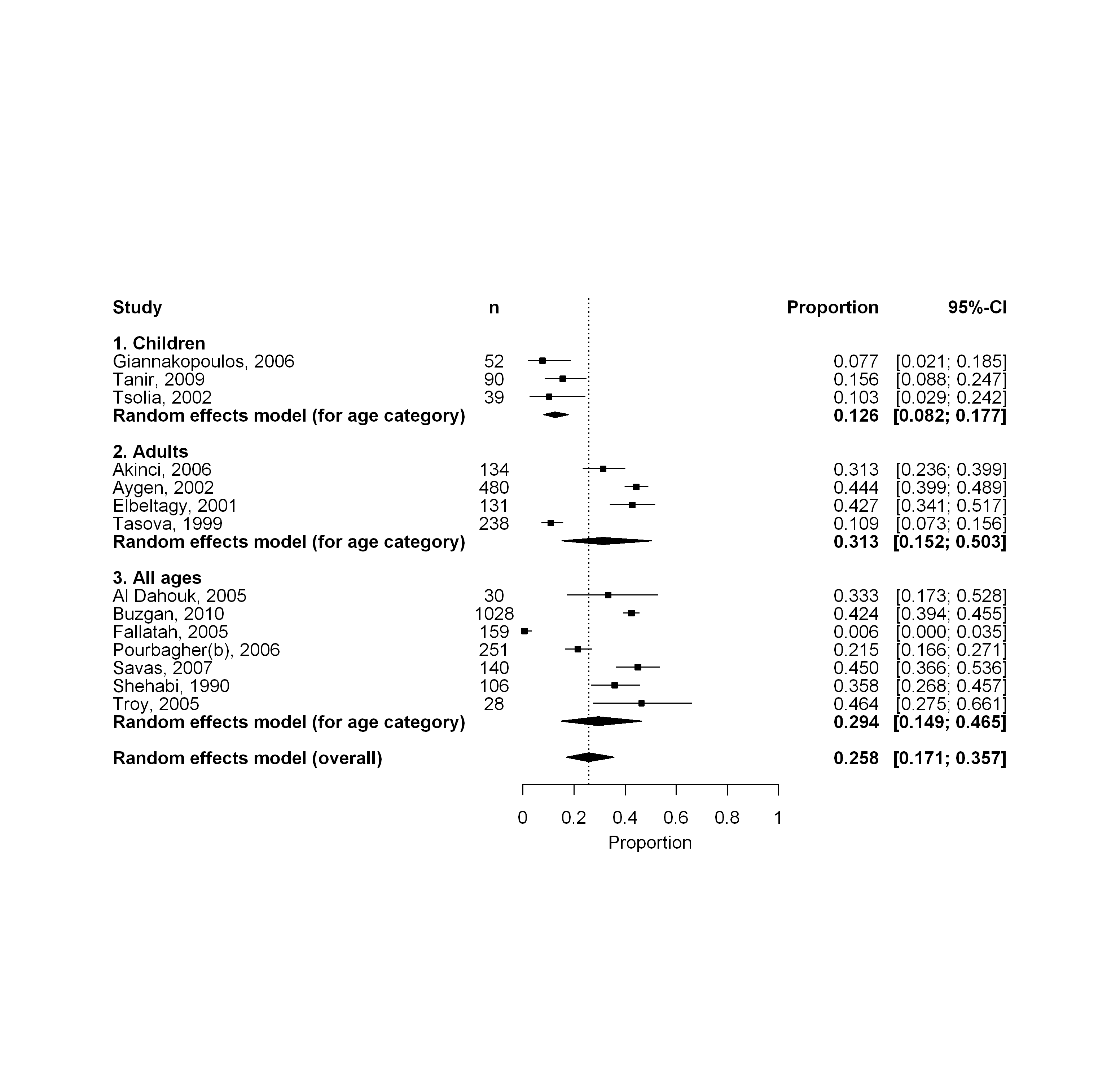

Supplement: Figure S8 — Forest plot for weight loss. (TIFF) [file pntd.0001929.s009.tiff]

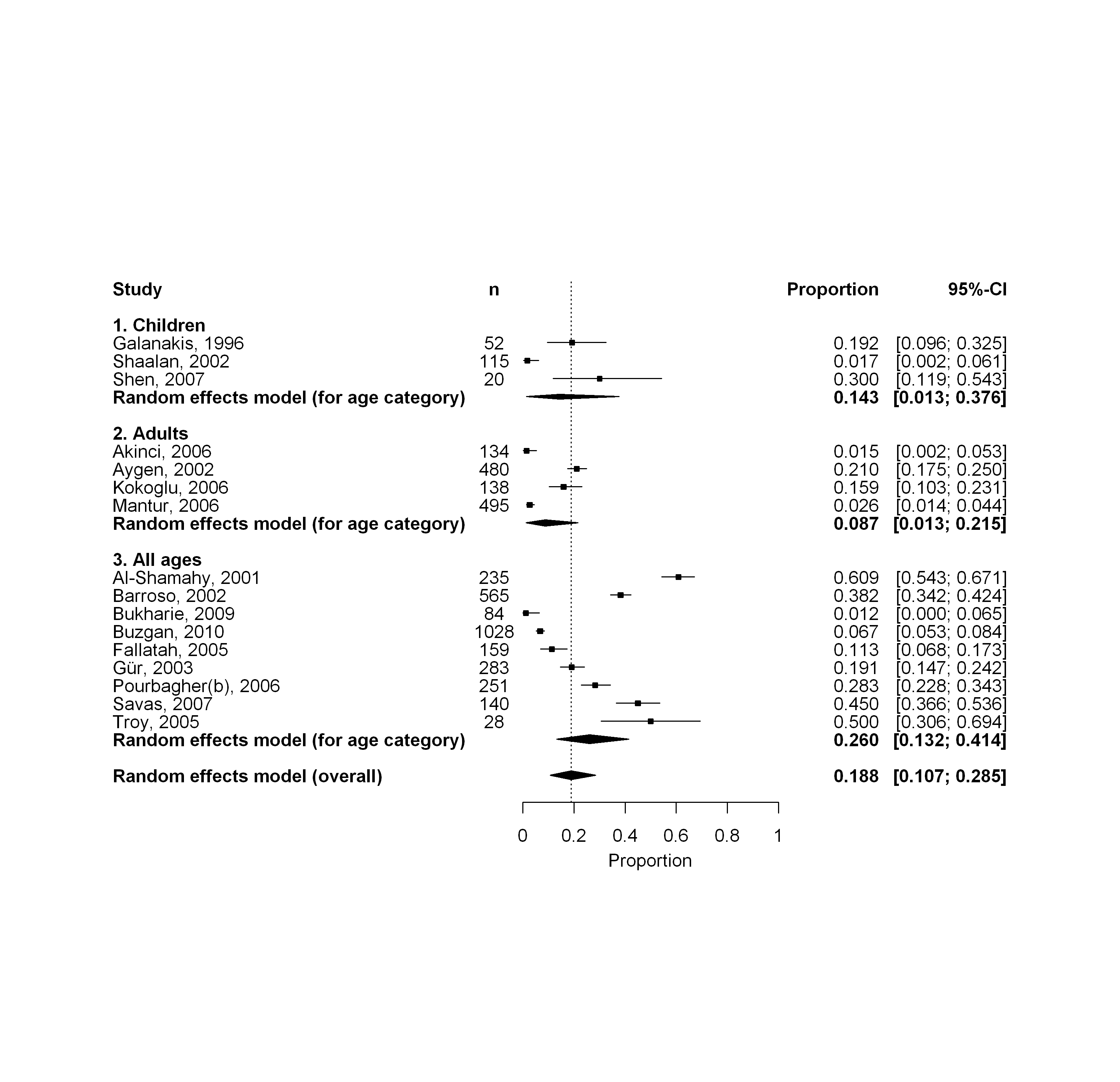

Supplement: Figure S9 — Forest plot for abdominal pain. (TIFF) [file pntd.0001929.s010.tiff]

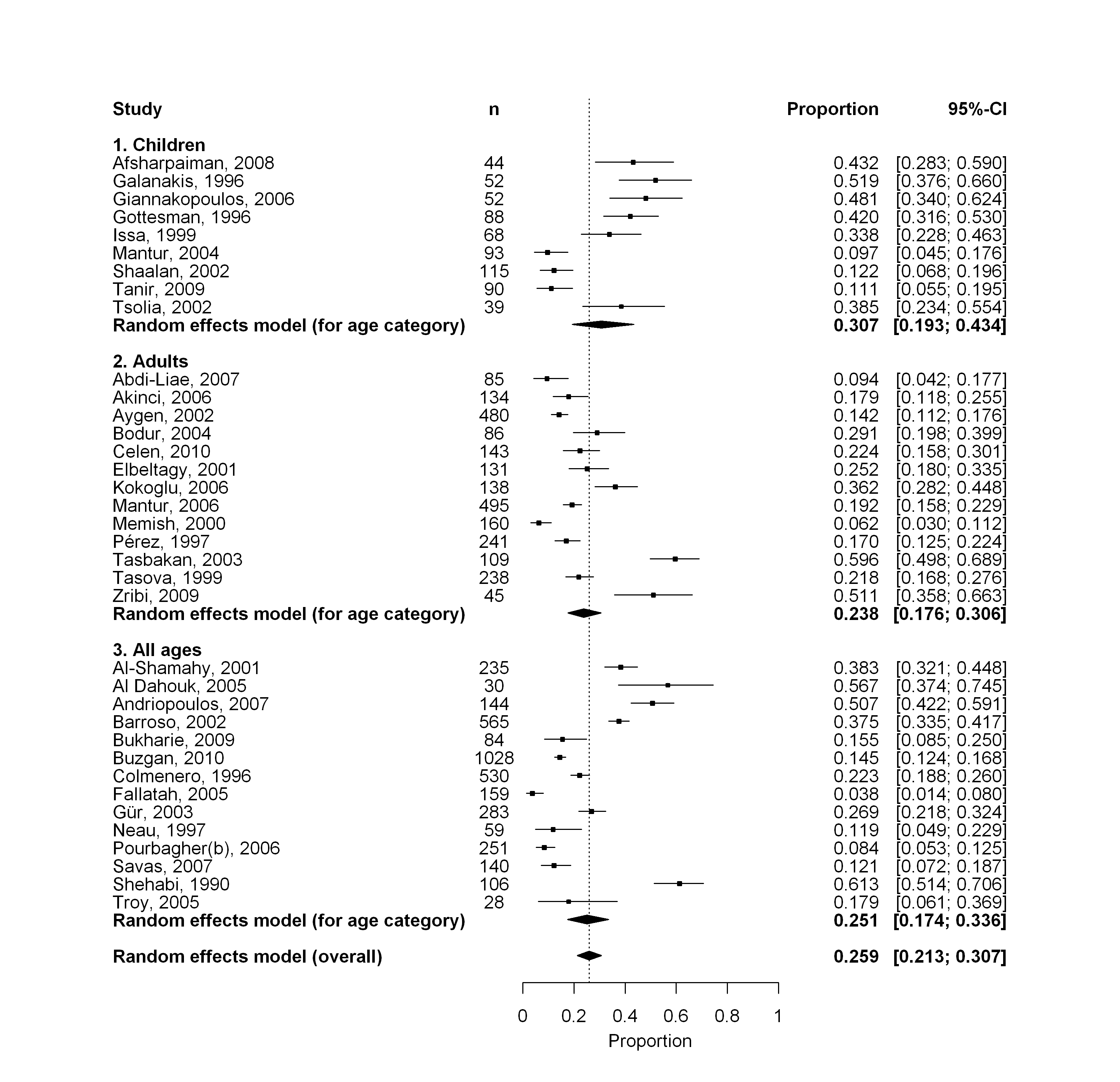

Supplement: Figure S10 — Forest plot for splenomegaly. (TIFF) [file pntd.0001929.s011.tiff]

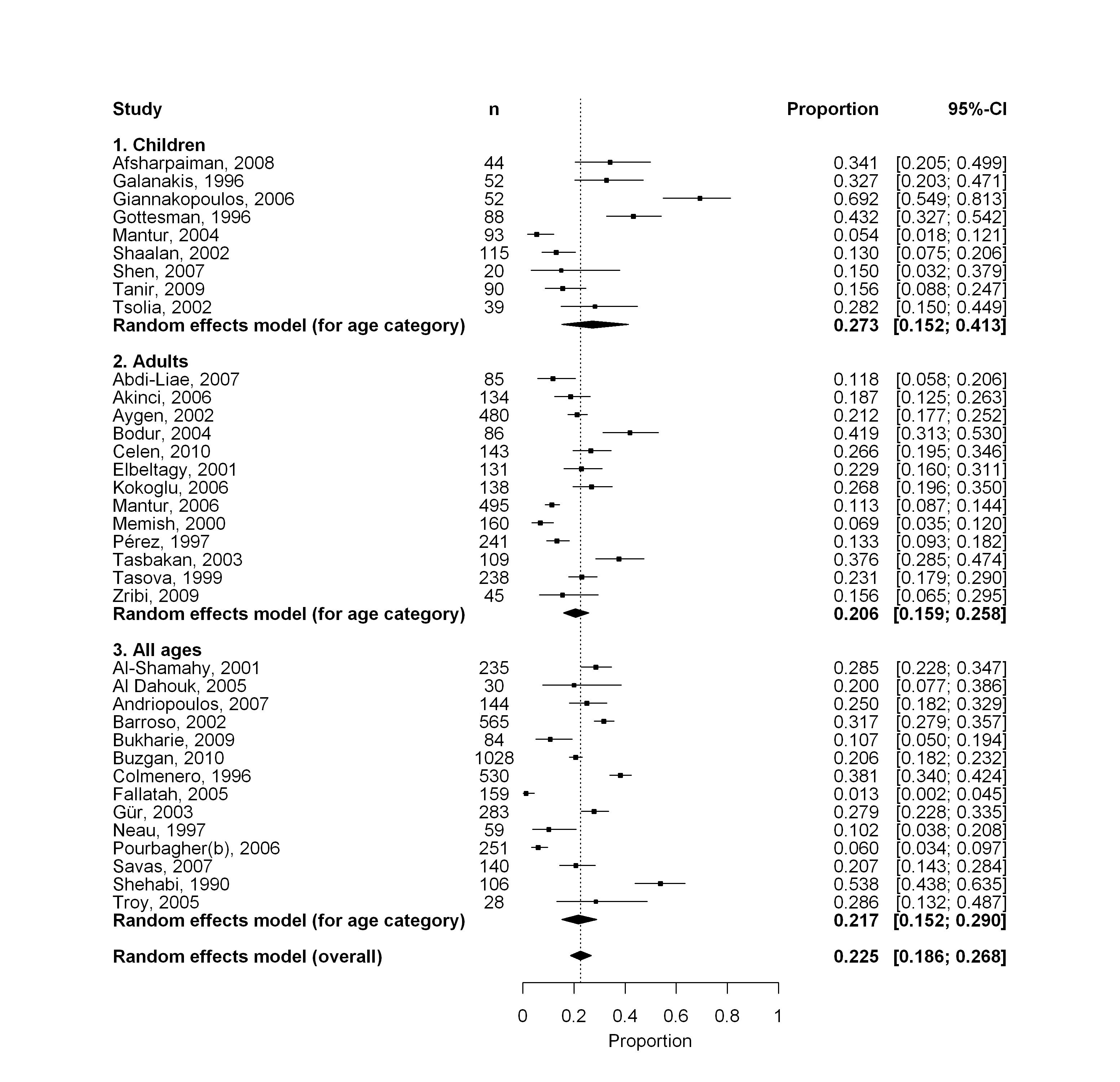

Supplement: Figure S11 — Forest plot for hepatomegaly. (TIFF) [file pntd.0001929.s012.tiff]

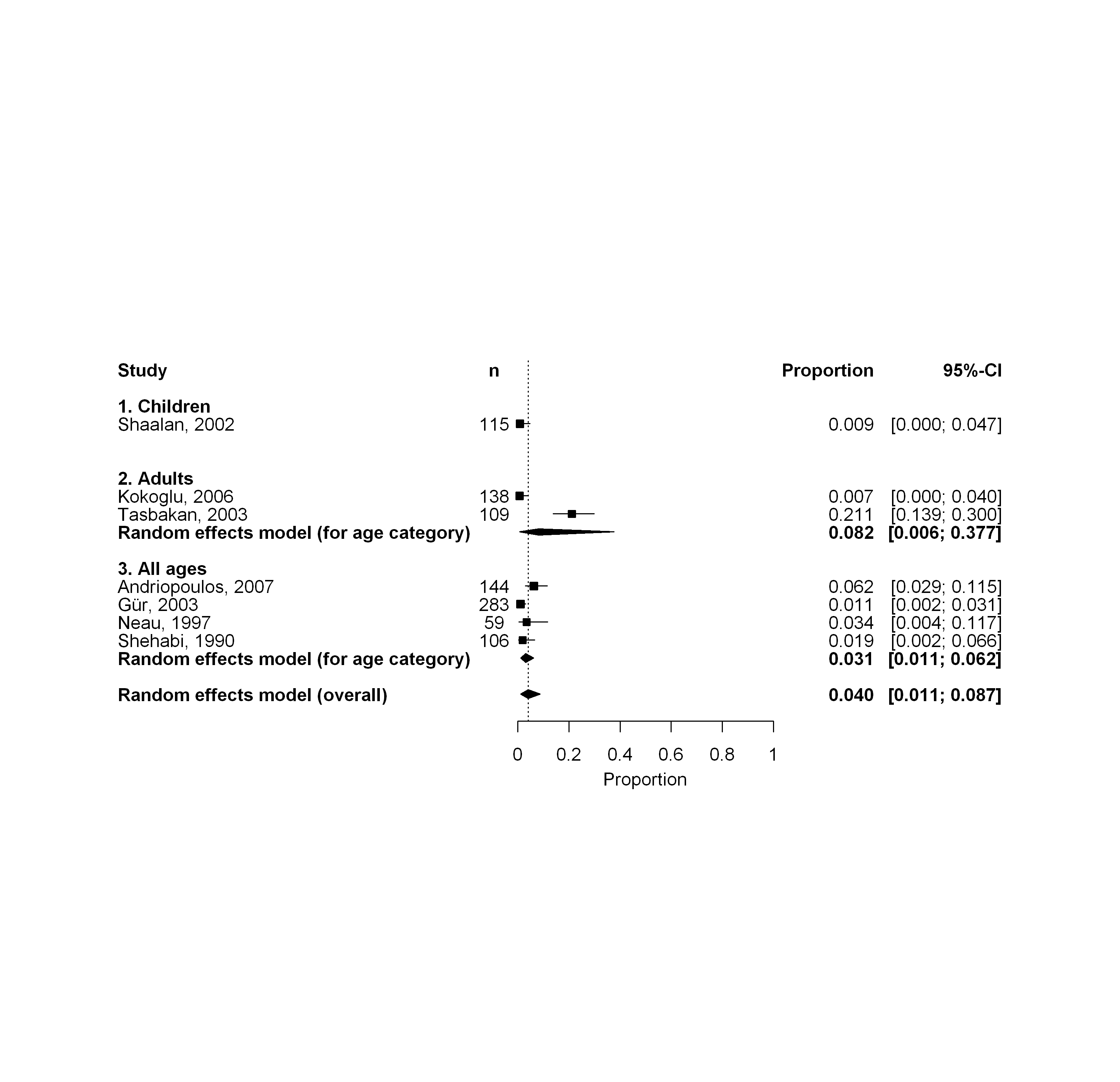

Supplement: Figure S12 — Forest plot for hepatitis. (TIFF) [file pntd.0001929.s013.tiff]

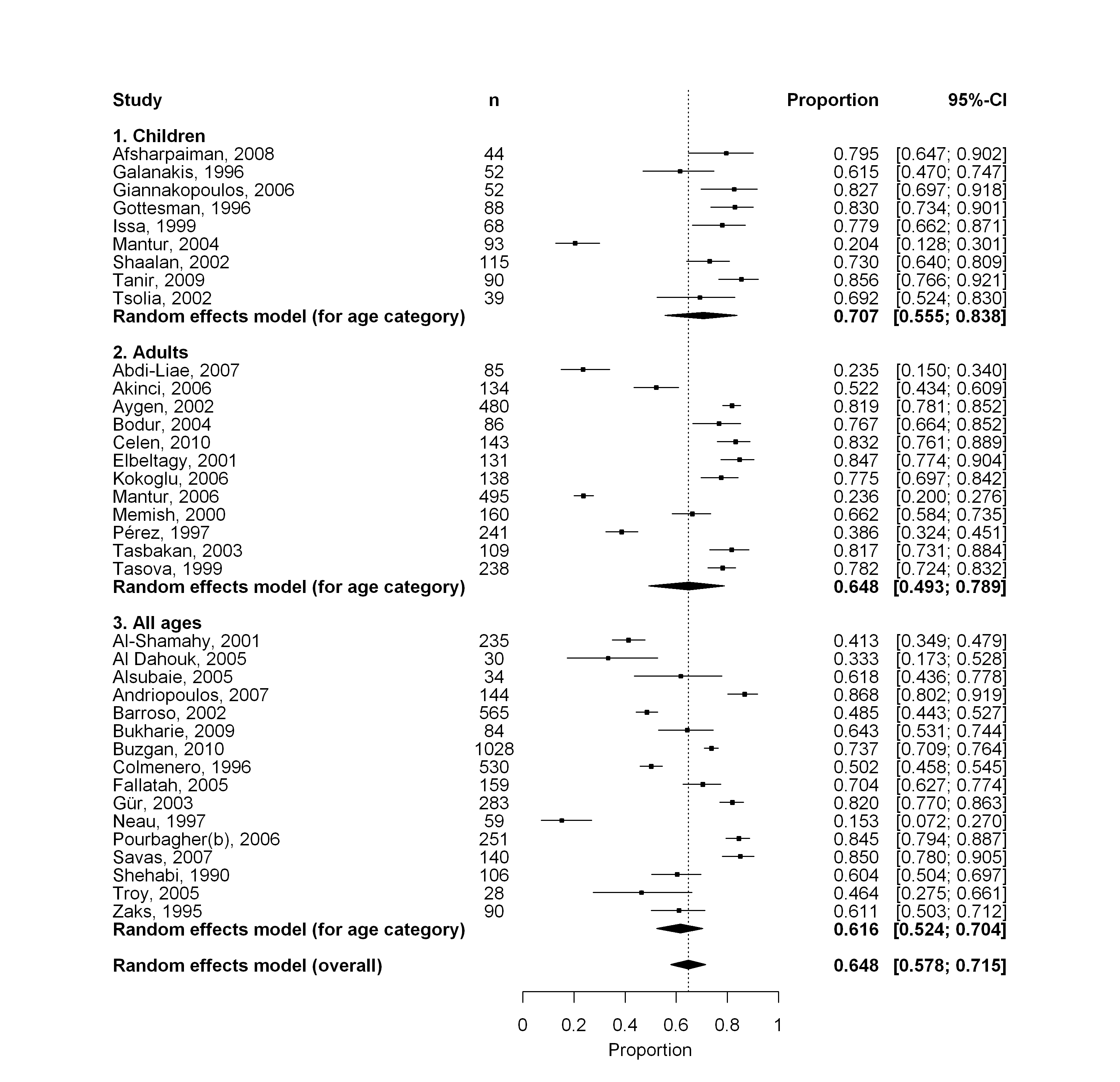

Supplement: Figure S13 — Forest plot for arthralgia. (TIFF) [file pntd.0001929.s014.tiff]

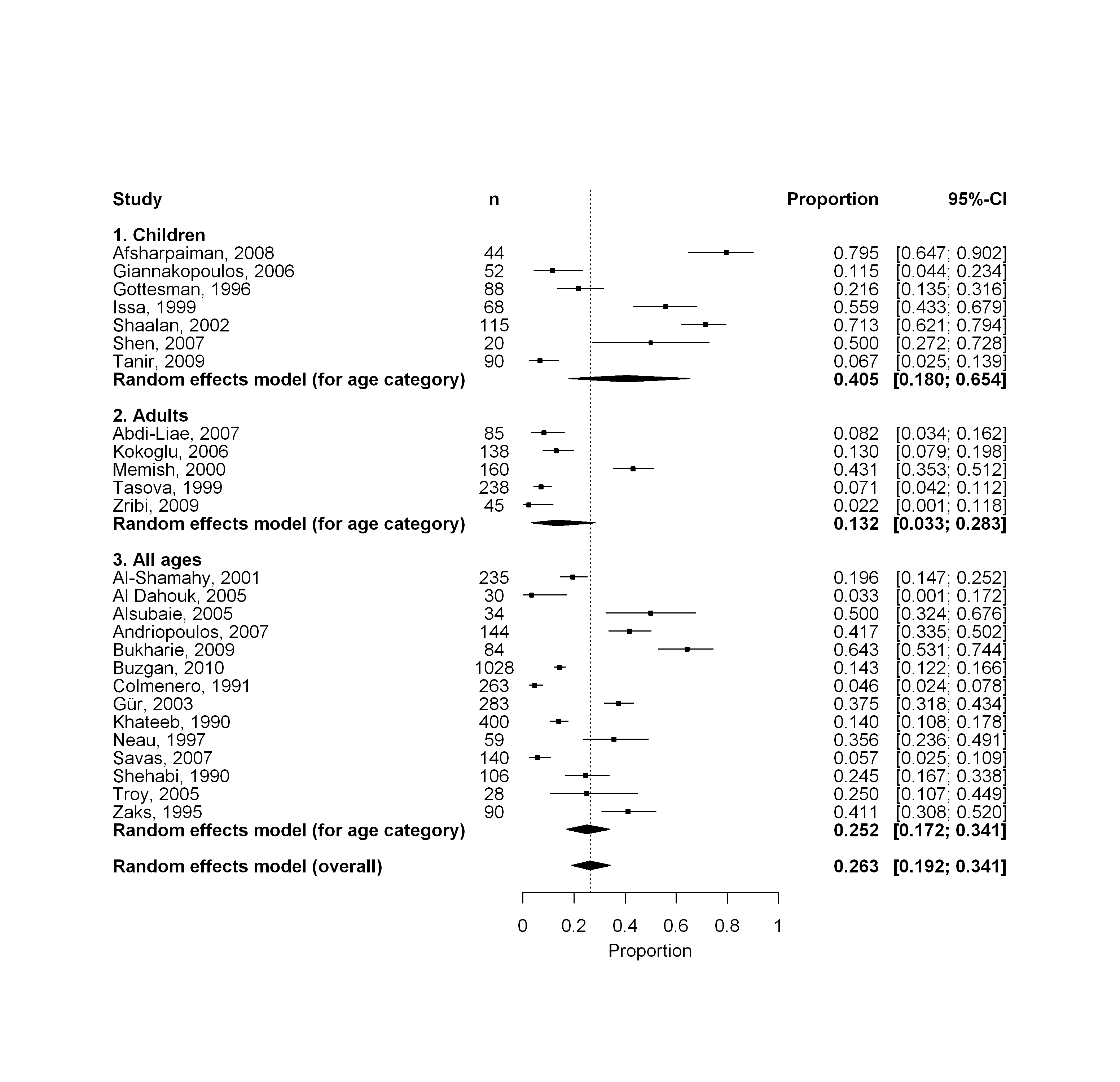

Supplement: Figure S14 — Forest plot for arthritis. (TIFF) [file pntd.0001929.s015.tiff]

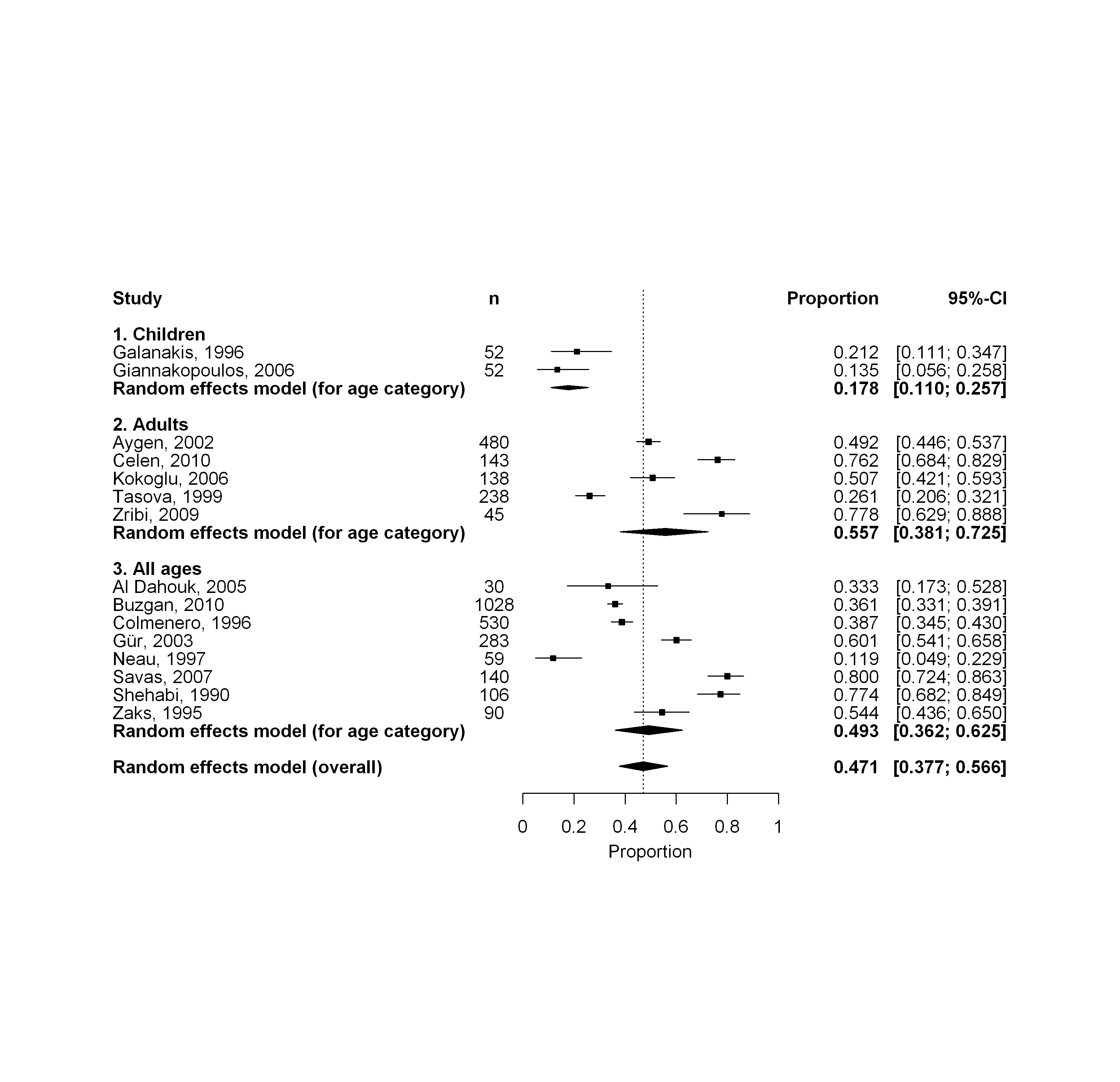

Supplement: Figure S15 — Forest plot for myalgia. (TIFF) [file pntd.0001929.s016.tiff]

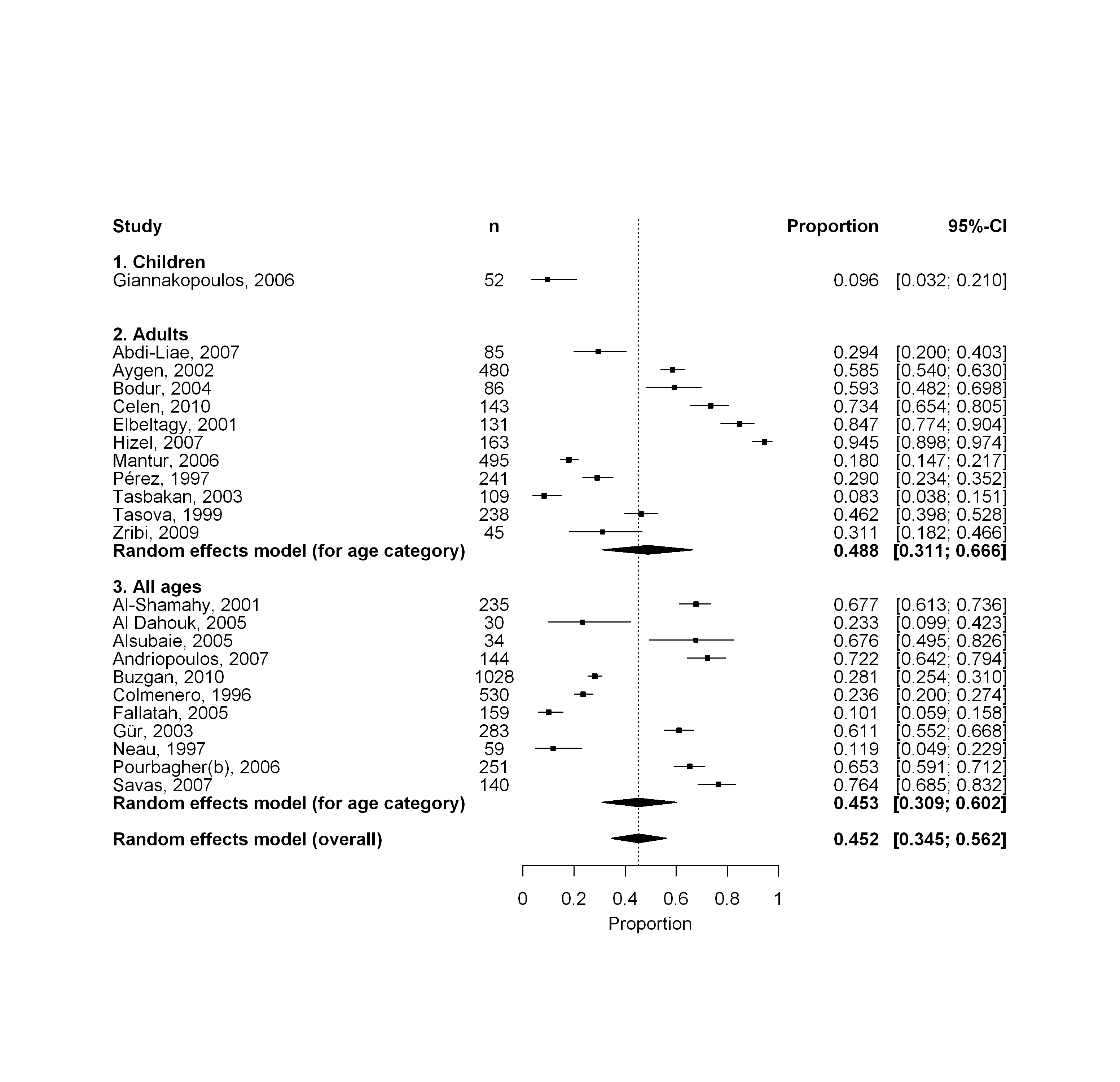

Supplement: Figure S16 — Forest plot for back pain. (TIFF) [file pntd.0001929.s017.tiff]

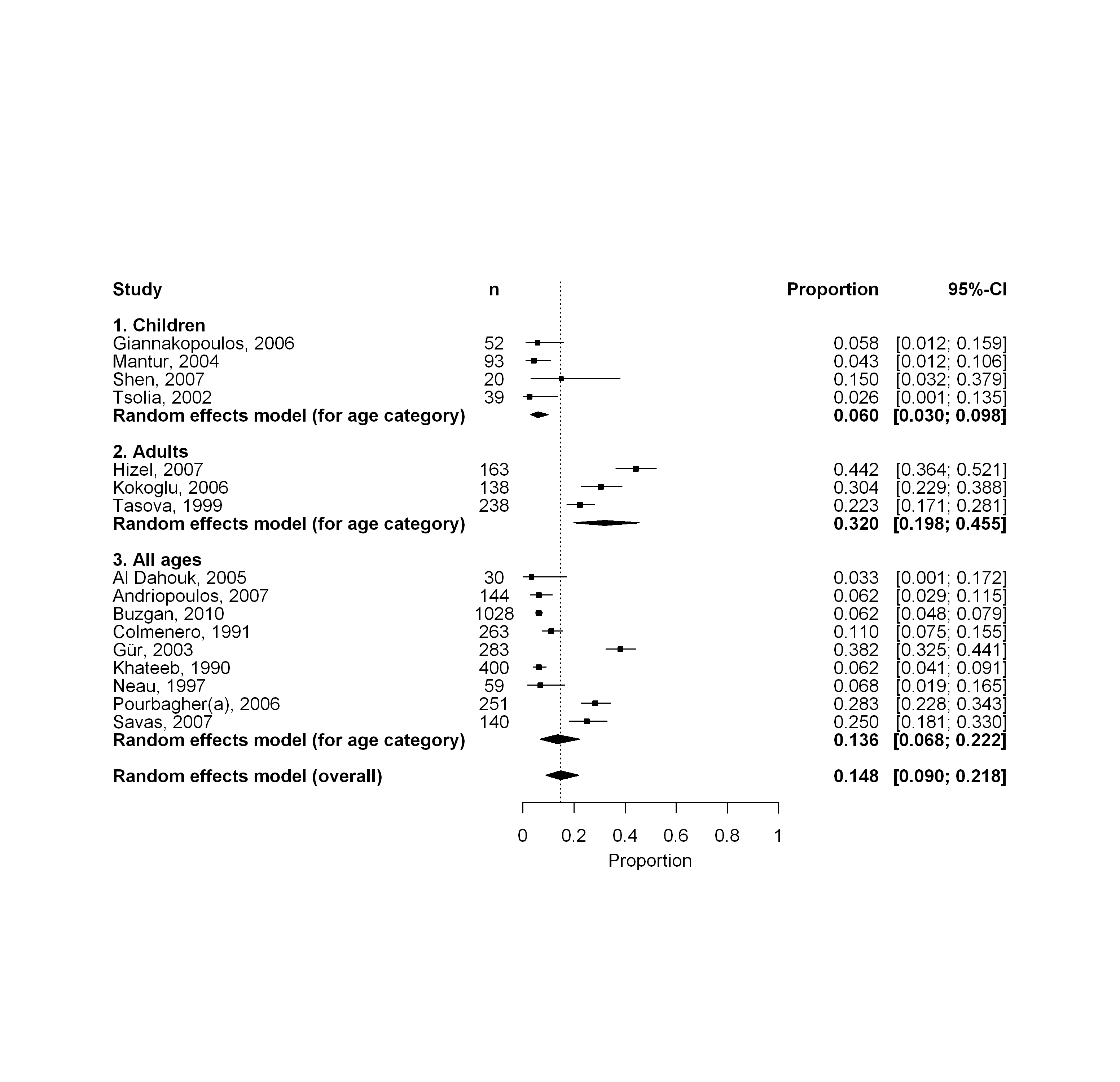

Supplement: Figure S17 — Forest plot for sacroiliitis. (TIFF) [file pntd.0001929.s018.tiff]

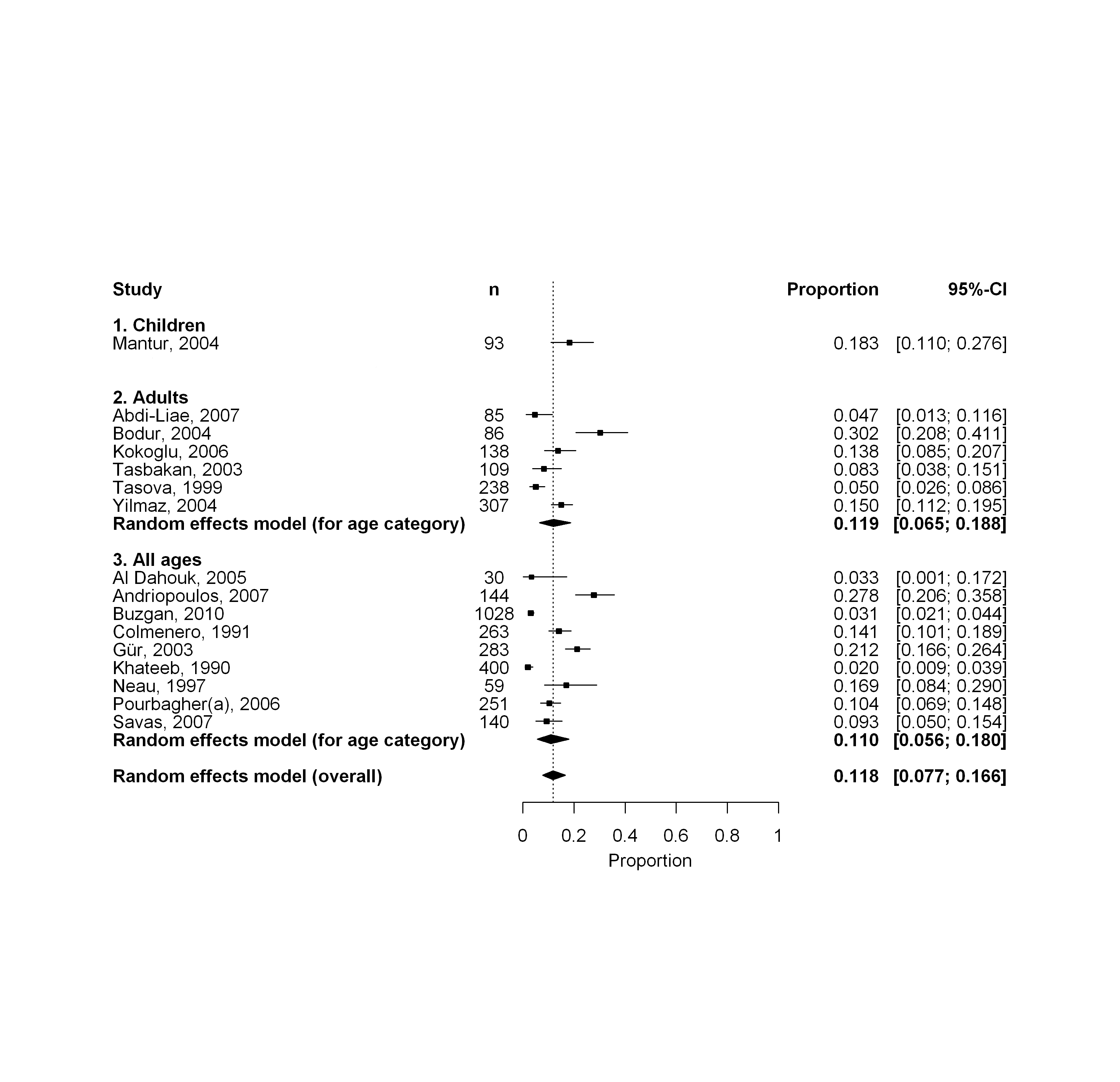

Supplement: Figure S18 — Forest plot for spondylitis. (TIFF) [file pntd.0001929.s019.tiff]

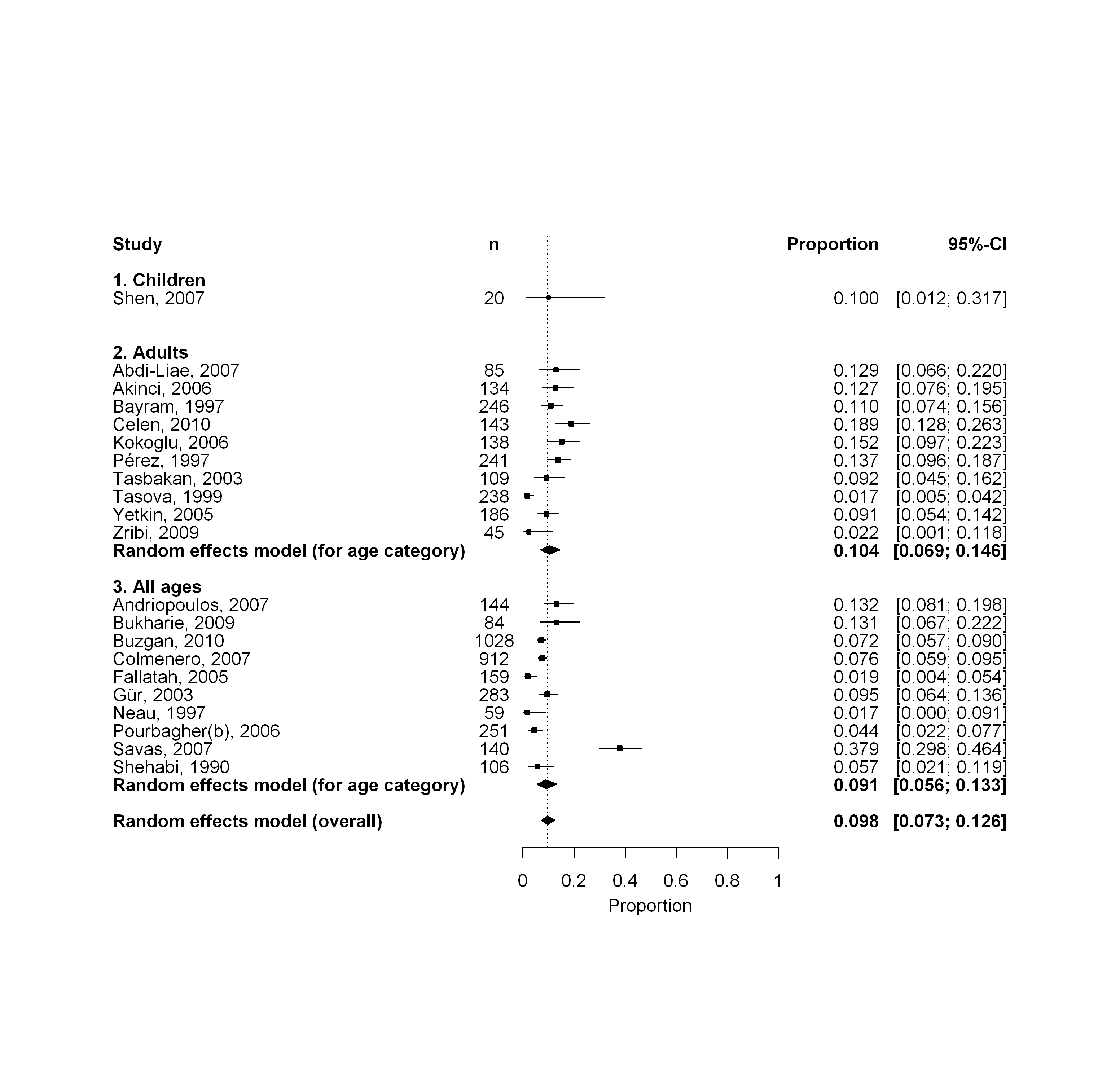

Supplement: Figure S19 — Forest plot for epididymo-orchitis. (TIFF) [file pntd.0001929.s020.tiff]

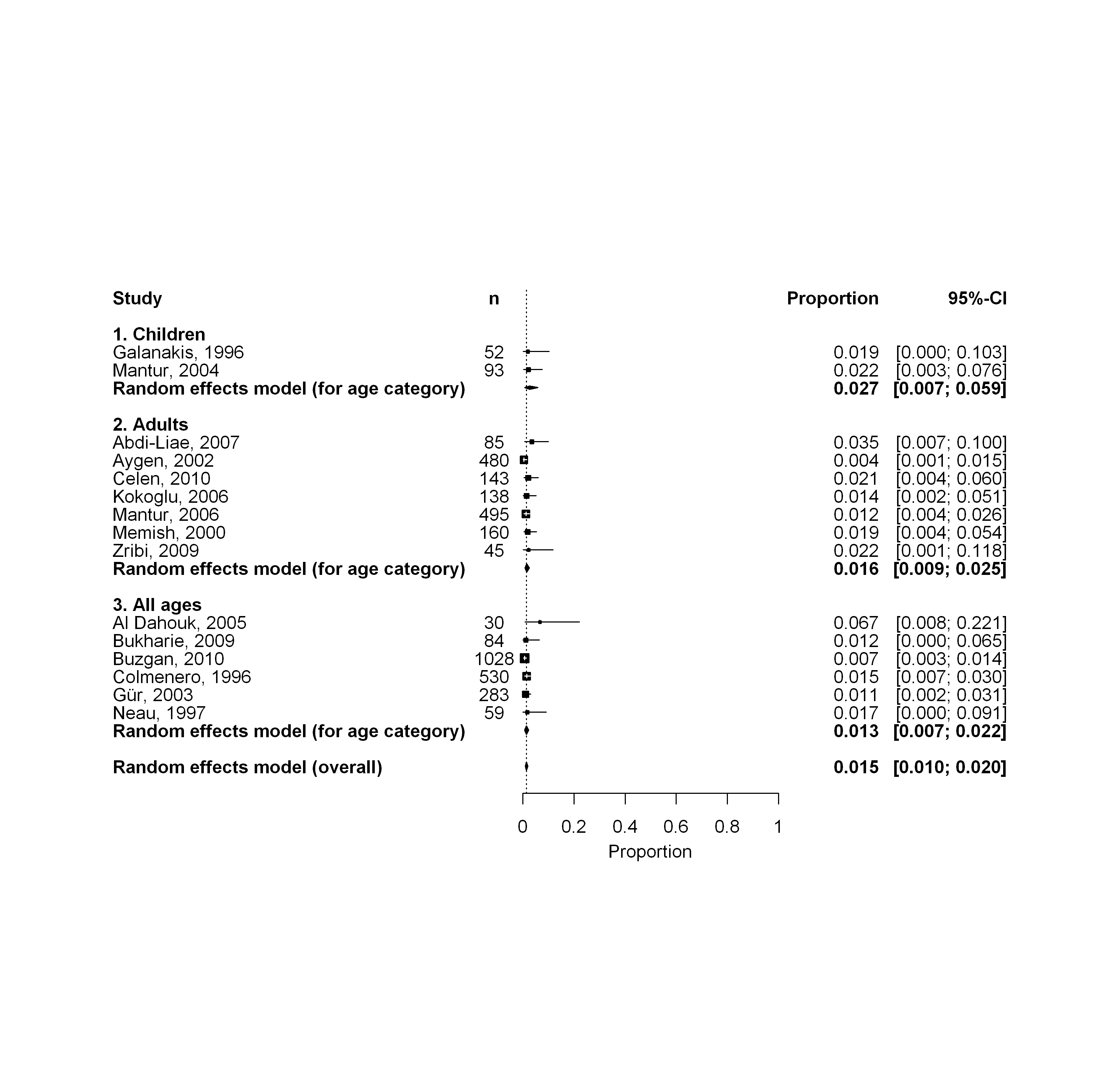

Supplement: Figure S20 — Forest plot for endocarditis. (TIFF) [file pntd.0001929.s021.tiff]

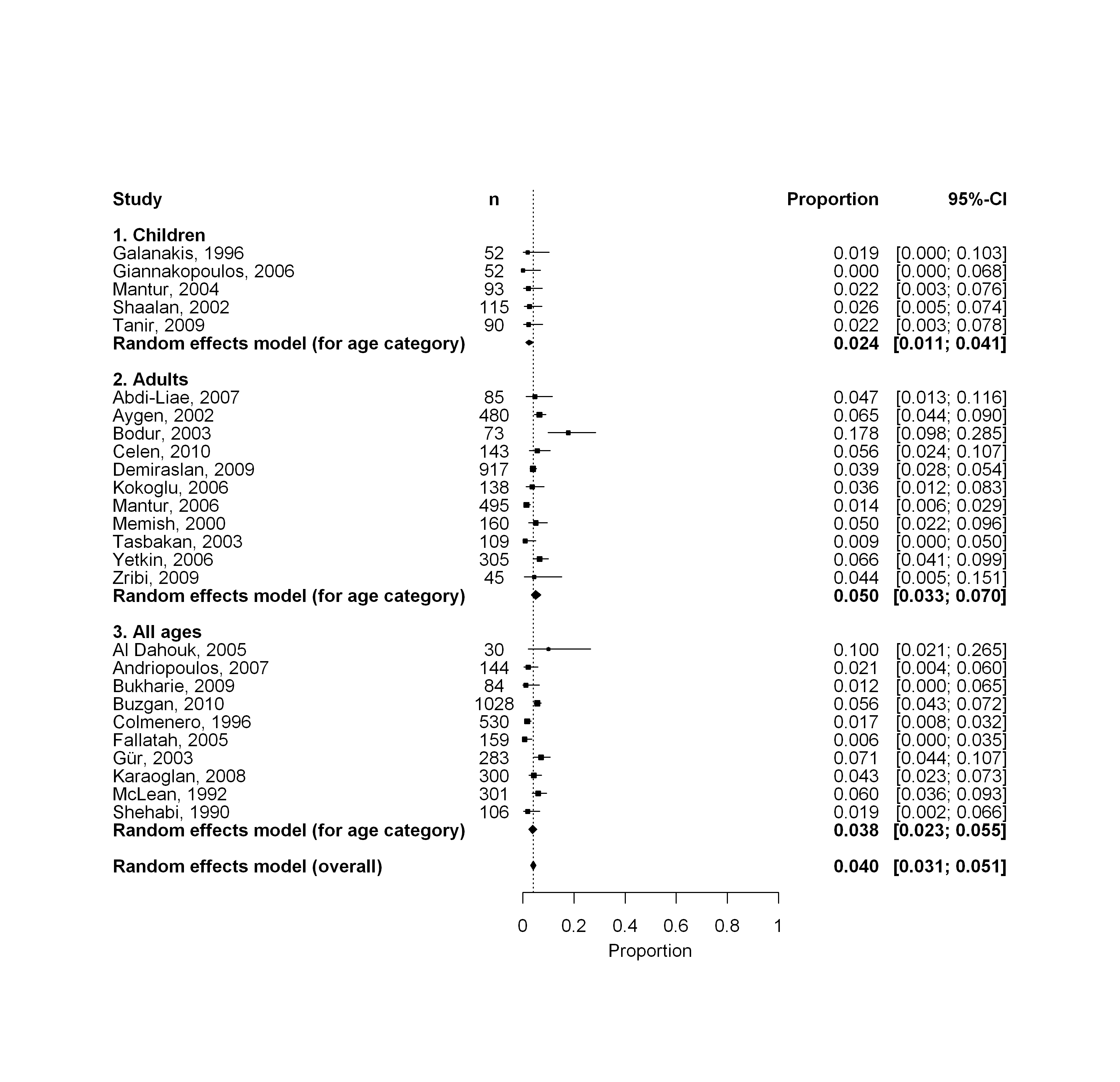

Supplement: Figure S21 — Forest plot for neurological sequelae. (TIFF) [file pntd.0001929.s022.tiff]

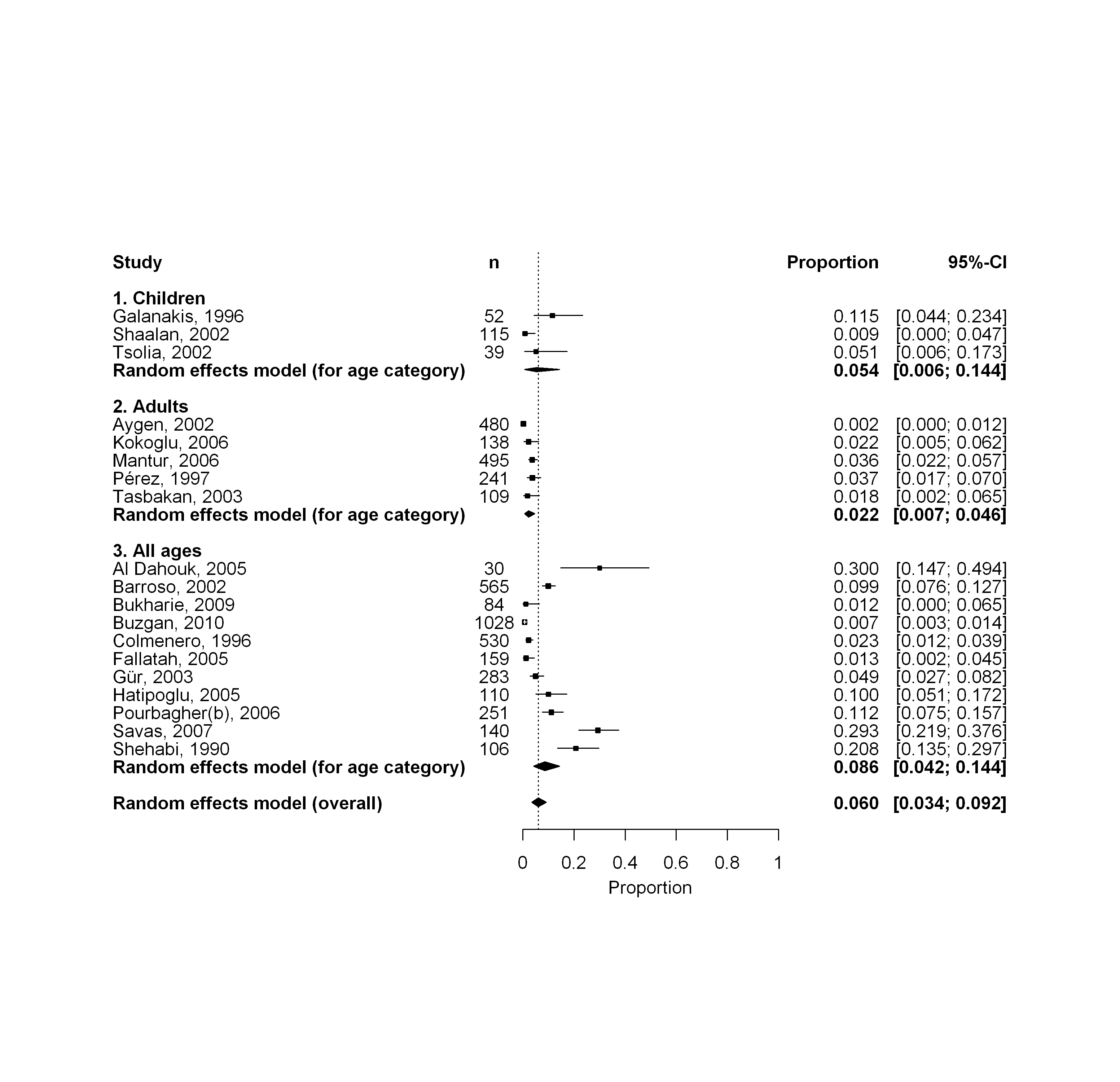

Supplement: Figure S22 — Forest plot for respiratory sequelae. (TIFF) [file pntd.0001929.s023.tiff]

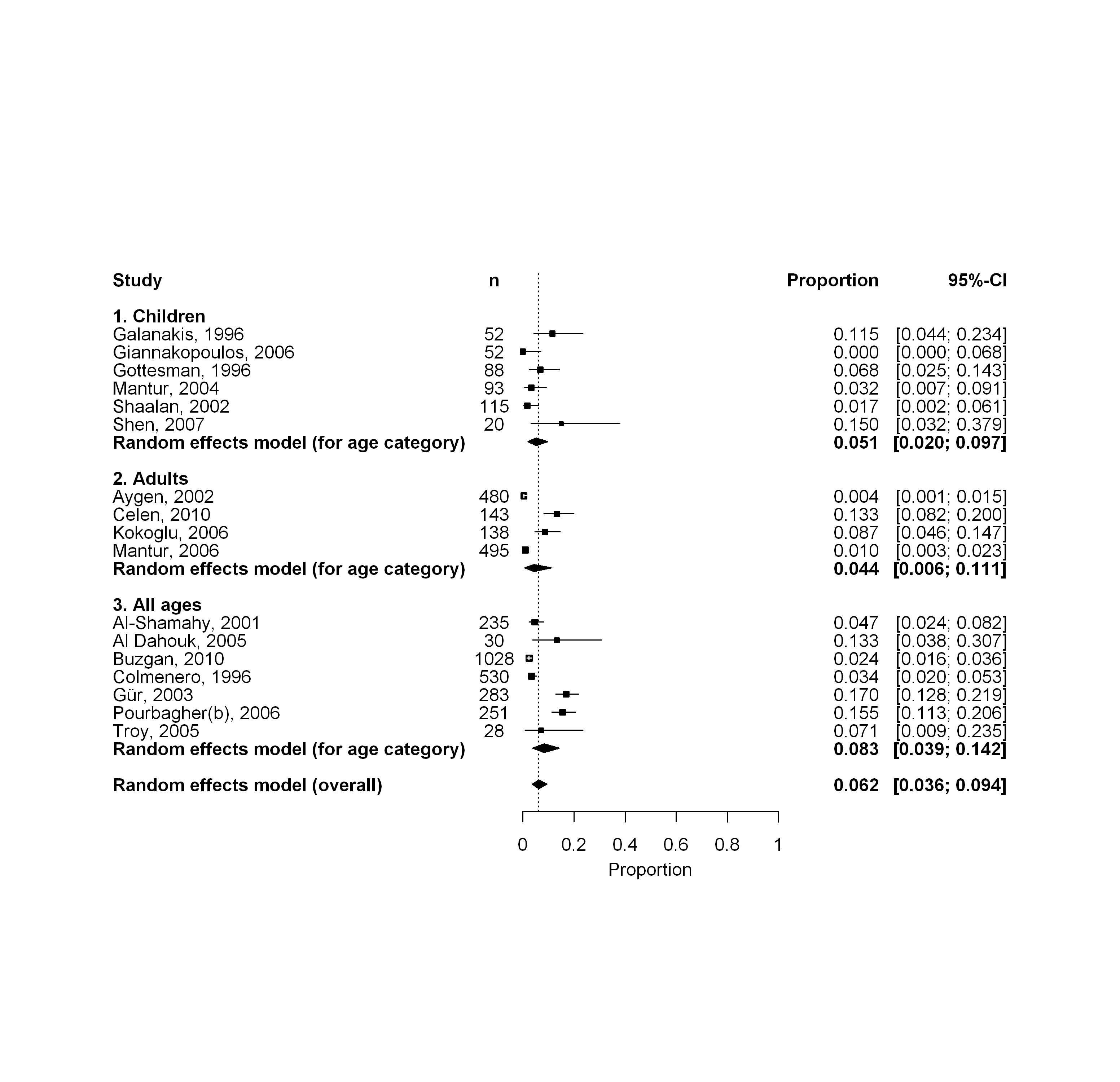

Supplement: Figure S23 — Forest plot for cutaneous sequelae. (TIFF) [file pntd.0001929.s024.tiff]
